# Cost-effectiveness analysis of colorectal cancer screening in Shanghai, China: A modelling study

**DOI:** 10.1016/j.pmedr.2022.101891

**Published:** 2022-07-04

**Authors:** Jie Wang, Lucie de Jonge, Dayna R. Cenin, Pei Li, Sha Tao, Chen Yang, Bei Yan, Iris Lansdorp-Vogelaar

**Affiliations:** a Department of Preventive Medicine and Health Education, School of Public Health, Fudan Health Communication Institute, Fudan University, Shanghai, China; b Erasmus MC, University Medical Center Rotterdam, Department of Public Health, Rotterdam, The Netherlands; c Centre for Health Services Research, School of Population and Global Health, The University of Western Australia, Perth, Western Australia, Australia; d The Center for Disease Prevention and Control Huangpu, Shanghai, China; e Shanghai Pudong New Area Center for Disease Control and Prevention, Shanghai, China; f Xi’an International Medical Center Hospital, Xi’an, China

**Keywords:** Colorectal cancer, Screening, Cost-effectiveness, Faecal immunochemical test, Risk assessment, China, Shanghai, CRC, colorectal cancer, FIT, faecal immunochemical test, ICER, incremental cost-effectiveness ratio, MISCAN-Colon, The Microsimulation Screening Analysis model for CRC, LYs, the number of life years, LYG, the number of life years gained, RA, risk assessment, ng Hb/mL, ng haemoglobin per mL buffer, µg Hb/g, µg haemoglobin per g faeces

## Abstract

**Background:**

The current community-based colorectal cancer (CRC) screening program in Shanghai, launched in 2013, invited individuals aged 50–74 years to triennial screening with a qualitative faecal immunochemical test (FIT) and questionnaire-based risk assessment (RA). We aimed to evaluate the effectiveness and cost-effectiveness of the existing Shanghai screening program and compare it to using a validated two-sample quantitative FIT.

**Methods:**

We simulated four strategies (no screening, Shanghai FIT, Shanghai FIT + RA and validated FIT) for the Shanghai screening program and evaluated CRC incidence, CRC mortality, the number of life years gained (LYG), the number of FITs, and colonoscopies required for each. An incremental cost-effectiveness analysis was performed to assess the cost- effectiveness of each strategy.

**Results:**

All screening modalities reduced CRC incidence and CRC mortality, gained extra number of LYG compared to no screening. Screening using the Shanghai FIT and validated FIT reduced CRC incidence from 45 cases to 43 per 1,000 simulated individuals (4.4%). Incidence was reduced to 42 cases (6.7%) using the Shanghai FIT + RA. All screening strategies reduced CRC mortality by 10.0% (from 10 to 9 deaths) and resulted in 6 to 7 LYG. The validated FIT was the most cost-effective among the evaluated strategies (ICER ¥26,461 per LYG).

**Conclusions:**

Our findings show that the current Shanghai screening program is (cost-) effective compared to no screening, but changing to a validated FIT would make the program more efficient.

## Highlights

• The current Shanghai CRC screening program is cost-effective.
• Changing to a validated FIT would make the program more efficient.
• The results were sensitive to an increase in the cost of the validated FIT.
• The results were sensitive to more participation in screening and colonoscopy.

## 1 Introduction

Colorectal cancer (CRC) is one of the most common cancers worldwide. (Sung et al., 2021) In recent years, CRC incidence in China has increased substantially from the historically low levels. (Pan et al., 2017) This rapid rise in incidence and the accompanying increase in disease burden is set to become a major public health challenge.

Screening for CRC has been implemented in many (largely high-income) countries and has contributed to reducing CRC incidence and mortality. (Schreuders et al., 2015) Shanghai is one of the first cities in China to implement a CRC screening program. (Li et al., 2019) Age-standardized CRC incidence in urban Shanghai has increased steadily between 1973 and 2015 rising from 13.58 per 100,000 person-years to 28.36 for males and from 11.92 to 22.33 for females. (Bao et al., 2016, Li et al., 2002) Initiated in 2013, the program targets individuals aged 50–74 years and offers triennial screening with a two-sample qualitative faecal immunochemical test (FIT) and a questionnaire-based risk assessment (RA).

While the RA plays an important role in CRC screening in China (Meng et al., 2009); it has led to a high false-positive rate in the Shanghai screening program. Moreover, although the program states that it utilizes a qualitative FIT with a positivity threshold of 100 ng haemoglobin per millilitre buffer (Gong et al., 2018) (ng Hb/mL, equivalent to 20 µg haemoglobin per g faeces (µg Hb/g) (Fraser et al., 2012), the actual cut-off has been shown to be between 1 and 5 µg Hb/g faeces in lab experiment. (Li et al., 2019) A lower cut-off increases test sensitivity, however, it also lowers specificity and increases the rate of false-positivity, which results in more unnecessary referrals for colonoscopy. This has been shown to impact compliance to diagnostic colonoscopy (Gong et al., 2018), thereby limiting the effect of screening.

The Shanghai qualitative FIT has been shown to have a low specificity (35%) and a high false-positive rate (65%) in the lab experiment (Li et al., 2019). Low compliance to follow-up colonoscopy has been identified as a significant challenge for CRC screening in China (Shanghai 28%, Pudong 22%). (Gong et al., 2018) Changing to a validated FIT, with a higher specificity, may help the screening program to overcome these issues.

These issues cast doubt on the effectiveness and cost-effectiveness of the current screening program. Therefore, this microsimulation modelling study aims to assess the effectiveness and cost-effectiveness of the Shanghai FIT and RA compared to a validated FIT.

## 2 Methods

### 2.1 Shanghai screening protocol

Detailed information on the CRC screening program in Shanghai has been provided elsewhere (Gong et al., 2018), also in the Supplementary Methods. In brief, the program was initiated in 2013 with individuals aged 50–74 years offered triennial screening with the Shanghai FIT (100 ng Hb/mL cut-off) and a RA. The RA involved a face-to-face interview consisting of nine questions including anorectal symptoms, related diseases (such as polyps and appendicitis), CRC family history, personal cancer history etc. (Gong et al., 2018) Positive individuals (either a positive Shanghai FIT result or a positive RA result) were recommended to have a diagnostic colonoscopy.

### 2.2 MISCAN-Colon

The Microsimulation Screening Analysis model for CRC (MISCAN-Colon) is a well-established microsimulation model developed at the Department of Public Health, Erasmus University Medical Center. (Loeve et al., 1999) The model has been extensively described previously and is described in Supplementary Methods. (Loeve et al., 1998, van Hees et al., 2015) For this study, we followed the approach developed by Gini et al. (Gini et al., 2021) and adjusted the MISCAN-Colon model to the situation in Shanghai. Data about age distribution and life expectancy of the Chinese population were direct inputs to the model. In order to match CRC incidence before the introduction of screening (Supplementary materials Figure S8), the onset of adenomas was adjusted. To match the stage distribution, transition probabilities for clinical diagnosis were adjusted. Stage distribution (Gong et al., 2015), localization of cancers in the colorectum (Shanghai, 2016) and five-year relative survival (Gong et al., 2015) after clinical diagnosis of a cancer are based on Chinese literature.

### 2.3 Screening strategies

We simulated a cohort of 100 million individuals born between 1939 and 1963 until death (restricted at age 100). The simulated individuals, aged between 50 and 74 years in 2013, were free of diagnosed CRC up to then and had a life expectancy as observed in China in 2010. (Guo, 2010) Individuals were screened triennially between ages 50–74. Screen-positive individuals were invited for a diagnostic colonoscopy. Surveillance was based on findings at diagnostic colonoscopy according to the European Society of Gastrointestinal Endoscopy (ESGE) Guidelines. (Hassan et al., 2013) We chose to simulate surveillance consistent with these guidelines because in China there is conflicting advice about the post-diagnostic colonoscopy pathway (including when to return to screening and the surveillance pathway). (Gong et al., 2017, Zhonghua et al., 2014, Chinese Society of Clinical Oncology, 2019, Endoscopology, 2015) Furthermore, the Asia Pacific Consensus Group did not provide precise guidelines on surveillance intervals, other than to suggest that such intervals should be tailored to the risk level. (Sung et al., 2015).

We assumed age-specific participation in screening and diagnostic colonoscopy based on the participation rates provided by Pudong CDC (Table 2). We assumed 80% adherence to surveillance colonoscopy. We assumed no difference in adherence to follow-up colonoscopy based on which of the two tests (FIT and/or RA) was positive. As there is no data available on adherence with rescreening in Shanghai, we assumed 90% of those who had previously participated would participate again, while 15% of those who had not participated in the previous round would now attend.

Under these conditions, we simulated three screening strategies: screening with the Shanghai FIT only, screening with Shanghai FIT + RA and screening using a validated two-sample FIT with a cut-off of 20 µg Hb/g faeces (validated FIT). In addition, we simulated a strategy without screening.

### 2.4 Test characteristics

We estimated the test characteristics of the Shanghai FIT and Shanghai FIT + RA so that the model predicted positivity and detection rates for advanced neoplasia are similar to those observed provided by the Pudong CDC in the first three years of screening in Pudong (2013–2015) (Table 1; Table 2; Supplementary Methods page 19 and Table S1).

**Table 1:** Positivity and detection rates obtained by estimation and provided by Pudong Centre for Disease Control for the first three years of screening (2013–2015).

	Positivity rate		Detection rate for non-advanced adenomas		Detection rate for advanced adenomas		Detection rate for CRC	
	Observed^a^ (95% CI)	Estimated^b^	Observed^c^ (95% CI)	Estimated^b^	Observed^c^ (95% CI)	Estimated ^b^	Observed^c^ (95% CI)	Estimated^b^
Shanghai FIT	0.145 (0.144 – 0.146)	0.145	0.025 (0.025 – 0.025)	0.025	0.018 (0.018 – 0.018)	0.018	0.004 (0.004 – 0.004)	0.004
Shanghai FIT + RA	0.231 (0.230 – 0.233)	0.231	0.038 (0.038 – 0.038)	0.038	0.026 (0.026 – 0.026)	0.026	0.005 (0.005 – 0.005)	0.005

Abbreviations: CRC, colorectal cancer; FIT, faecal immunochemical test; RA, risk assessment; CI, confidence interval.

a The observed positivity rate is determined as the total number of positive tests divided by the total number of participants using the specific screen test. In case of the Shanghai FIT + RA, this screen test was considered positive when the Shanghai FIT and/or the risk assessment were positive.

b The estimated positivity and detection rates are obtained by the Nelder-Mead Simplex method (Nelder and Mead, 1965) as explained in the methods section.

c The observed detection rates were corrected for lack of adherence with colonoscopy to allow unbiased comparison with estimated detection rates. This is established by multiplying the observed positivity rate with the positive predictive value.

**Table 2:** Test characteristics, age-specific participation rates and costs associated with colorectal cancer screening and treatment.

**Test characteristics (%)**				
	Shanghai FIT^a, b^	Shanghai FIT + RA^a, b^	Validated FIT	Colonoscopy
Sensitivity small adenoma (≤5mm)^c^	0.0	0.0	0.0	75.0
Sensitivity medium adenoma (6–9 mm)^c^	8.7	9.4	7.1	85.0
Sensitivity large adenoma (≥10 mm)^c^	20.3	33.0	46.9	95.0
Sensitivity CRC early preclinical^c^	44.6	74.2	66.0	95.0
Sensitivity CRC late preclinical^c^	78.9	93.1	90.0	95.0
Specificity	87.4	79.3	96.7	86.0
Complications of colonoscopy by perforation^d, e^				0.012
**Age-specific participation (%)**	Shanghai FIT/Shanghai FIT + RA/Validated FIT		Diagnostic colonoscopy after positive Shanghai FIT	Diagnostic colonoscopy after positive Shanghai FIT + RA
50–54	12.6		33.1	24.3
55–59	24.8		35.8	26.9
60–64	40.1		35.3	27.3
65–69	61.7		31.4	24.0
70–74	52.8		28.5	21.7
FIT rescreening	90.0		NA	NA
**Costs (2019 Chinese Renminbi Yuan (¥))^f^**			**Probabilistic sensitivity analysis, ranges^p^**	
			*Gamma-distribution*	
Per validated FIT^g^	25.00		[12.50; 50.00]	
Per Shanghai FIT^h^	13.00		[6.50; 26.00]	
Per RA^i^	3.48		[1.74; 6.96]	
Per positive screening test^j^	15.00		[7.50; 30.00]	
Per colonoscopy^k^	375.30		[187.65; 750.60]	
Per polypectomy	654.83		[327.42; 1309.66]	
Per perforation of colonoscopy^m^	19761.04		[9880.52; 39522.08]	
Treatment by stage and location^n^				
Stage I CRC	35227.92		[17613.96; 70455.84]	
Stage II CRC	37342.58		[18617.29; 74685.58]	
Stage III CRC	37481.16		[18740.58; 74962.32]	
Stage IV CRC	38472.04		[19236.02; 76944.08]	
General outpatient cost^o^	23.30		[11.65; 46.60]	

Abbreviations: CRC, colorectal cancer; FIT, faecal immunochemical test; RA, risk assessment; NA, not applicable.

a It was assumed that the probability a CRC bleeds and thus the sensitivity of a FIT for CRC depends on the time until clinical diagnosis (Lansdorp-Vogelaar et al., 2009).

b Specificity and sensitivity based on the positivity rates and detection rates of advanced neoplasia observed in the first screening round in Pudong, Shanghai. This data for this was provided by Pudong Centre for Disease Control. Sensitivity for adenomas smaller than 5 mm was assumed to be 0% for all tests.

c Different sensitivities are defined in the model as it simulates the development of colorectal cancer through the adenoma carcinoma sequence. As each simulated person ages, one or more adenomas may develop and these adenomas can progress in size increasing from small (<5 mm) to medium (6–9 mm) to large (greater than10 mm). Some adenomas can develop into preclinical cancer, which may progress through cancer stages I to IV.

d Complications are conditional on polypectomy, and we assume that polypectomy is only performed if colonoscopy is positive. A complication is considered as an unplanned hospital admission within 30-days of a colonoscopy.

e Rate of perforation is based on data from Shanghai, China, 2014 (Shi et al., 2014).

f Costs are from a health system perspective and do not include patient time costs. All costs are presented in Chinese Renminbi Yuan (¥) and are indexed to 2019 prices (Tool, 2019).

g Costs for two-sample quantitative FIT provided by Pudong Centre for Disease Control and are based on the current reimbursement funding arrangement.

h Cost for a two-sample FIT used in the Shanghai screening program taken from Gong and colleagues, 2018 (Gong et al., 2018).

i Cost of the risk assessment provided by Pudong Centre for Disease Control.

j These costs are provided to encourage those with positive screening test to attend diagnostic colonoscopy, as well as support other activities related to colonoscopy. Costs provided by Pudong Centre for Disease Control.

k Costs for colonoscopy are based on sources from China (Wang et al., 2012) and includes cost of bowel preparation (Huang et al., 2017).

l Costs polypectomy is based on sources from China (Wang et al., 2012) and includes costs of biochemical and pathological testing (Huang et al., 2017). This cost is in addition to the cost for colonoscopy.

m Costs for perforation during colonoscopy are based on sources from China (Wang et al., 2012).

n Costs of cancer treatment are taken from the Chinese setting. These costs were for the hospitalization stage and the first year after CRC has been diagnosed. It included medicine, surgical, examination, and treatment fee. It didn’t include surveillance (CT scan, blood test, endoscopy, etc.) after initial treatment of CRC (Shanghai, 2016, Wu et al., 2014).

o Co-payment made by patients when seeing a doctor and undergoing a colonoscopy (Shanghai, 2016).

p Ranges of 95% confidence intervals for the costs in the probabilistic sensitivity analysis were obtained by halving and doubling the base case values. Using these ranges, the shape parameter k and the scale parameter θ are calculated as input for the Gamma-distributions.

To estimate the validated FIT characteristics, we modelled the Dutch trial according to the approach of Goede and colleagues. (Goede et al., 2013) Here, the test characteristics of the validated FIT were fitted to the positivity and detection rates of advanced neoplasia observed in the first screening round of two Dutch randomised trials, which utilised the OC-Sensor micro (Eiken Chemical, Tokyo, Japan). (Hol et al., 2010, Hol et al., 2009, van Rossum et al., 2008, van Roon et al., 2011) (Table 2) The obtained sensitivity and specificity were then transferred to the mode for China. The characteristics differ to those previously presented as the natural history of the MISCAN-Colon model has been updated since this publication. (Rutter et al., 2016).

The test characteristics for all FITs were adjusted to take into account the effect of individuals without adenomas who always test positive and adenomas that do not bleed (systematic false-positive and false-negative results). (van der Meulen et al., 2016) The Shanghai FIT + RA and the validated FIT were considered as one single test in the MISCAN Colon model. The test characteristics of colonoscopy are based on a systematic review of polyp miss rates in tandem colonoscopy studies. (van Rijn et al., 2006) (Table 2). The lack of specificity of colonoscopy reflects the detection of benign hyperplastic polyps, which are not cancer precursors. (Schroy et al., 2013).

### 2.5 Costs of screening, surveillance and CRC care

Costs were included from the healthcare sector perspective. The costs of the Shanghai FIT and the RA were provided by Pudong CDC. The Pudong CDC also provided the costs for the validated FIT based on the current reimbursement funding arrangement. These costs include the test kits, their distribution, return, analysis, and expenses in marketing. Costs for colonoscopy, polypectomy and complications from colonoscopy were obtained from research based in China (Table 2). (Wang et al., 2012, Li et al., 2016) Costs for cancer care were based on costs of cancer treatment in the Chinese setting; (Wu et al., 2014) and the cost of treating advanced CRC (Stage IV) came from expert consultation (Shanghai, 2016) All costs are presented in Chinese Renminbi Yuan (¥), standardised to 2019 prices using the consumer price index. (Tool, 2019).

### 2.6 Outcomes

For all strategies, the model estimated CRC incidence, CRC mortality, benefits (the number of life years (LYs) and the number of life years gained (LYG)), burden (number of screening tests, and diagnostic and surveillance colonoscopies required), harms (number of colonoscopy complications and false-positive tests), as well as total costs (Renminbi (¥)). A false-positive test is defined as a positive screening test followed by a colonoscopy with no clinical findings. Costs and LYs were discounted using a standard annual rate of 3%. Undiscounted results and results discounted to 5% are presented in Supplementary Results Tables and Figures.

### 2.7 Cost-effectiveness analysis

To determine the cost-effectiveness of all strategies, strategies were rank-ordered according to their costs. Strategies that cost more than (a combination) of other strategies while gaining fewer LYs were considered inefficient. For the remaining strategies, cost-effectiveness was expressed by the incremental cost-effectiveness ratio (ICER) as incremental cost per LYG compared to the next less effective strategy. The willingness-to-pay (WTP) threshold was set at three times the Chinese gross domestic product per capita in 2018 (¥193,931 Chinese Renminbi Yuan, equal to $29,313US) per LYG. The strategy with the highest ICER below the WTP threshold was considered as the most efficient strategy.

### 2.8 Sensitivity analysis

We conducted six sensitivity analyses to assess the robustness of our results. First, due to the uncertainty about the cost of the validated FIT, we explored the impact of varying its cost by assuming a 50% reduction and a two-fold increase. Second, we adjusted the treatment costs for stages II-IV because of the limited availability of cost data and the possibility that the assumed costs may not reflect the actual costs of treating CRC. Treatment costs were adjusted to reflect the proportional increase in lifetime health care costs for the different stages of CRC. (Lang et al., 2009) The costs for stage I remained unchanged from the base case scenario. Third, as there is currently no available information on quality of life in the Chinese setting, they were excluded from the main analysis. Therefore, we conducted a sensitivity analysis utilising international quality of life measurements (Supplementary Methods Table S2). (Ness et al., 1999) Fourth, we assessed the impact of an alternative surveillance pathway, derived from Chinese literature (Supplementary Methods Figure S7). (Gong et al., 2017, Zhonghua et al., 2014) Fifth, in order to determine the effect of improved participation in screening and diagnostic follow-upon outcomes, the participation was increased to 60% and 80%, respectively. Finally, due to uncertainty in performance of the Shanghai tests, we calibrated the FIT and risk questionnaire characteristics based on data published about CRC screening in Guangzhou, China. (Lin et al., 2019).

### 2.9 Probabilistic sensitivity analysis

In the probabilistic sensitivity analysis, we assessed the uncertainty of the costs to evaluate future economic improvements and change in health care costs. For every strategy, we performed 1,000 simulations each containing different costs drawn from a gamma distribution (Table 2).

### 2.10 Ethic compliance

This study met the institution’s guidelines for protection of human subjects concerning their safety and privacy.

## 3 Results

### 3.1 Base case

After adjustment to the Shanghai population, the MISCAN-Colon model predicted that, without screening, lifetime CRC incidence and mortality would be 45 and 10 per 1,000 individuals, respectively (Table 3). Introducing screening reduced both CRC incidence and mortality in all three screening strategies. Screening with the Shanghai FIT and the validated FIT reduced CRC incidence from 45 to 43 cases per 1,000 individuals (4.4% decrease) and to 42 cases (6.7% decrease) with the Shanghai FIT + RA. All screening strategies reduced CRC mortality by 10.0% (to 9 deaths per 1,000 individuals).

**Table 3:** Costs and effects (discounted at 3%) per 1,000 simulated individuals for all screening strategies.

**Screening strategy**	**Primary screening test episodes^a^**	**Colonoscopies**	**False Positives**	**Complications^b^**	**CRC Incidence**	**CRC Mortality**	**Life Years Gained^c^**	**Total Costs (¥)**	**ICER (¥)**
No Screening	0	45	0	0.01	45	10	0.00	1,080,042	
Shanghai FIT	2,145	171	56	0.01	43	9	6.19	1,129,839	8,045
Shanghai FIT + RA	2,142	197	70	0.01	42	9	6.62	1,146,176	Extended Dominated
**Validated two sample FIT**	**2,150**	**122**	**14**	**0.01**	**43**	**9**	**6.97**	**1,150,479**	**26,461**

Note: Screening in Pudong occurs every three years between ages 50 to 74.

Bold highlights the most efficient screening strategy under the willingness-to-pay threshold.

Abbreviations: CRC, colorectal cancer; FIT, faecal immunochemical test; RA, risk assessment; ICER, incremental cost-effectiveness ratio.

a Shanghai FIT + RA and the validated two-sample FIT were both considered to be one single test episode in the simulation.

b Due to rounding and the low probability of an adverse event (complication) during a colonoscopy (0.012%), the number of complications per 1,000 individuals simulated did not change between different screening strategies.

c Life years gained compared to a situation without screening.

The number of screening episodes used in the three different strategies was comparable (ranging from 2,142 to 2,150). As would be expected, colonoscopy demand increased with the introduction of screening. The validated FIT had the lowest colonoscopy demand (122 colonoscopies per 1,000 individuals.), followed by the Shanghai FIT (171 colonoscopies) and the Shanghai FIT + RA (197 colonoscopies). The number of false-positive tests was substantially lower for the validated FIT (14 tests) compared to the Shanghai FIT (56 tests) and the Shanghai FIT + RA (70 tests).

**Fig. 1:**
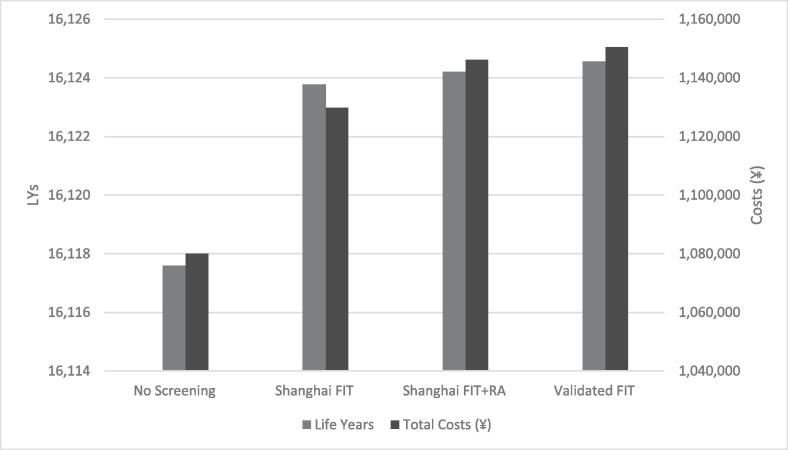
Costs and life years (discounted at 3%) per 1,000 simulated individuals of all colorectal cancer screening strategies and a strategy without screening. Abbreviations: FIT, faecal immunochemical test; LYs, life years; RA, risk assessment.

The validated FIT screening strategy had the largest LYG (6.97 per 1,000 individuals), followed by Shanghai FIT + RA (6.62) and Shanghai FIT (6.19) (Fig. 1, Table 3). Without screening the cost of diagnosing and treating CRC was ¥1,080,042 per 1,000 individuals. Screening increased costs by ¥49,797 to ¥70,436 (4.7 to 6.6%). The current screening program (Shanghai FIT + RA) cost ¥146,1768, an increase of ¥66,134 (6.2%). At the WTP threshold (¥193,931/LYG), the most efficient screening strategy was the validated FIT with an ICER of ¥26,461 per LYG. The strategy using the Shanghai FIT had a lower ICER (¥8,045 per LYG (Table 3)), but was less effective than the validated FIT. The strategy using the Shanghai FIT + RA was less costly and less effective than the validated FIT and thus extended dominated.

### 3.2 Sensitivity analysis

Our results were robust to changes in the discounting rates, producing similar results without discounting (Supplementary Results Table S1) and under a discounted rate of 5% (Supplementary Results Table S2). In addition, they were robust to the reduction in the cost of the validated FIT (Supplementary Results Table S3a), the adjustment of the treatment costs (Supplementary Results Table S3b), the use of international quality of life measurements (Supplementary Table S3c), the derived Chinese surveillance pathway (Supplementary Results Table S3d), and the test characteristics based on data from Guangzhou, China (Supplementary Table S3e). Under all these assumptions, the validated FIT remained the most efficient strategy. However, when the cost of the validated FIT was increased by 200% (Supplementary Table S3e), the ICER of the validated FIT exceeded the WTP threshold. In addition, when participation for screening and diagnostic follow-up was increased, the ICER of the Shanghai FIT + RA was the highest below the WTP threshold (Supplementary Table S3g). Therefore, in these two scenarios, screening with the Shanghai FIT + RA was the most cost-effective strategy.

### 3.3 Probabilistic sensitivity analysis

The probabilistic sensitivity analysis suggests that at the WTP threshold of ¥193,931, the optimal screening test (the validated FIT) is the cost-effective strategy in more than 75% of the 1,000 considered cost values (Supplementary Results Figure S4). The existing program was not cost-effective in any of the 1,000 values for the costs.

## 4 Discussion

This study investigated the effectiveness and cost-effectiveness of three CRC screening strategies (Shanghai FIT, Shanghai FIT + RA, and a validated FIT), using the MISCAN-Colon model. Our results suggest that all strategies were almost equally effective at reducing CRC incidence and mortality and resulted in similar LYG. The strategy utilising the validated FIT had substantially lower harms, however it cost more than the alternatives. At the WTP threshold of ¥193,931 Renminbi (equivalent to $29,313 US) per LYG, triennial screening between ages 50–74 with the validated FIT at a cut-off 20 µg Hb/g faeces was the most cost-effective strategy. Although our results demonstrate that the current screening program is not the most cost-effective, it is encouraging that even with low levels of participation screening reduces CRC incidence and mortality. In order to improve the cost-effectiveness and reduce the burden of screening, the program could shift to a validated FIT.

The effectiveness of screening is impacted by the characteristics of the screening test(s). Although in theory the Shanghai FIT and the validated FIT have the same cut-off value, the two tests have different characteristics. The validated FIT has higher specificity and acceptable sensitivity, resulting in less false-positives and lower colonoscopy demand. The estimated complications and CRC mortality were comparable with the other screening strategies, and CRC incidence was the same as with Shanghai FIT, and only slightly less than with Shanghai FIT + RA.

Better specificity is an important explanation for the better cost-effectiveness of the validated FIT. The Shanghai FIT, both with and without RA, has low estimated specificity (87.4% and 79.3%, respectively). This is consistent with the reported low specificity of qualitative FITs (75.1%) in China. (Wu et al., 2014) The low specificity and therefore high false-positivity of the Shanghai FIT may be a consequence of unstandardized cut-off of the test. This, coupled with visual interpretation of the results (rather than a numerical result) which introduces inter-observer variation, influenced the number of referrals for colonoscopies. This supports our suggestion to move to the validated FIT.

Although the cost-effectiveness of CRC screening in high incidence countries is widely accepted, less is known about the cost-effectiveness in lower incidence countries like China. Previous research has shown a wide range of screening strategies to be cost-effective, with some being cost saving, in Western countries. (Ran et al., 2019) Modelling studies using a Markov model found CRC screening to be cost-effective in the Chinese population (Cai et al., 2016, Wang et al., 2020, Liang et al., 2019, Huang et al., 2014), which is consistent with our findings. The study demonstrating the cost-effectiveness of a stool-based test combined with a RA over a stool-based test alone are hard to compare to this study, because they investigated different screening strategies (especially for surveillance) and the assumptions of the population and test characteristics were different. (Huang et al., 2014) To the best of our knowledge, there are no cost-effectiveness analyses investigating the use of a quantitative FIT in China until now. Studies outside of China found that in population-based screening, the likely total cost of qualitative FIT was greater than an automated high-volume quantitative FIT, since quantitative FIT provide the tools to control colonoscopy referrals and for most health systems the cost of the screening test is smaller than colonoscopy. (Allison et al., 2014).

Our results were sensitive to improved participation in screening and diagnostic follow-up and an increase in the cost of the validated FIT. With increased participation in screening and diagnostic colonoscopy, the Shanghai FIT + RA became the most cost-effective strategy. However, under this strategy the colonoscopy demand became very high (635 per 1,000 simulated individuals), almost three times as high as with the validated FIT. Considering that colonoscopy capacity is limited in many regions in China (Huang et al., 2016), it is very likely that the Chinese health system does not have the capacity to satisfy such high-intensity colonoscopy demand. Colonoscopy demand is a bottleneck in CRC screening. When the demand exceeds the available capacity, the implementation of the screening program will be obstructed and the effectiveness of screening will be influenced. (Arrospide et al., 2018) Therefore, any proposal for CRC screening that increased colonoscopy demand would not be feasible with the current available resources in China. In a complementary article (Cenin et al., 2022), annual testing using the validated one-sample FIT, with a cut-off of 10 µg Hb/g from ages 45–80 years would be the most cost-effective, while still requiring fewer colonoscopies than the existing triennial Shanghai screening strategies. If Shanghai was to move to a validated FIT, the large purchasing power would likely reduce the cost from our estimated ¥25. Our sensitivity analysis showed the validated FIT to be highly cost-effective under a 50% reduction in this cost.

The CRC screening participation rates in China are considerably lower than in other countries. (Yang et al., 2020) Compared with participants who were at least positive with the Shanghai FIT, participants with only a positive RA were the least likely to undergo colonoscopy. (Gong et al., 2018, Cheng et al., 2018, Wu et al., 2019) This suggests low public confidence in RA, probably due to its high false-positive rate. (Gong et al., 2018) Although previous studies showed that RA improved sensitivity and helped to identify individuals with non-bleeding lesions (Meng et al., 2009), more research is needed to develop and validate optimal RA tools. In Japan, a risk score including sex, age, CRC family history, BMI, and smoking history was shown to be more effective and cost-effective than colonoscopy and FIT. (Sekiguchi et al., 2020) In Hong Kong, a similar risk scoring system which included self-reported diabetes has been effectively used to prioritize high-risk individuals for colonoscopy and polypectomy when there is limited colonoscopy capacity. (Wong et al., 2014).

This study has four limitations. First, the impact of the existing Shanghai screening program may have been overestimated. We used data from the first three years of screening (2013–2015) under the assumption that, given the triennial screening interval, this data would represent the prevalent screening round. As prevalent screening rounds generally have a higher yield than subsequent rounds, the calibrated test characteristics are likely to be overestimated. However, even with these overestimates our results demonstrate that the Shanghai FIT is inferior to the validated FIT. In addition, as some Chinese surveillance guidelines recommend individuals return to screening one year after a negative diagnostic colonoscopy, there may be some contamination in the data. However, as so few individuals participated in diagnostic colonoscopy (6.37% in Pudong), we believe that this is unlikely to affect our results. Second, the Shanghai FIT + RA was modelled in MISCAN-Colon as one single screening test episode. This approach ensures that potential correlation between both tests is appropriately taken into account. However, it is also likely that the outcomes of such questionnaires are correlated over screening rounds, which was not accounted for. Third, as there are conflicting surveillance guidelines for CRC screening in China we modelled surveillance according to the ESGE Guidelines. (Hassan et al., 2013) When we modelled Chinese surveillance guidelines our results did not change. Finally, although the model is adjusted to China, the underlying model structure is based on the European population. We assumed the same progression of adenoma to cancer in the model for China as in the Dutch model. As there may be differences between these populations, long-term impacts of screening may not be generalizable.

Based on this modelling study, CRC screening in Shanghai is highly cost-effective in reducing the CRC burden and shifting to a validated FIT could improve the cost-effectiveness. In addition, using the validated FIT, the quantitative value of faecal haemoglobin concentrations is known and it is therefore possible to adjust the positive thresholds to match the desired positive rate and colonoscopy referrals in different social contexts. (Symonds et al., 2018).

In conclusion, our findings show that the Shanghai CRC screening program is cost-effective. This study supports the continuity of the program and highlights switching to the validated FIT to increase its efficiency.

## Funding

This study is funded by Research Grant for Health Science and Technology of Pudong Health and Family Planning Commission of Shanghai, China (Grant No.PW2017A-7). This research benefitted from our participation in the National Cancer Institute’s Cancer Intervention and Surveillance Modeling Network (CISNET) (grant number: U01‐CA199335).

## CRediT authorship contribution statement

**Jie Wang:** Conceptualization, Formal analysis, Methodology, Software, Validation, Visualization, Writing – original draft, Writing – review & editing. **Lucie de Jonge:** Conceptualization, Formal analysis, Methodology, Software, Validation, Visualization, Writing – original draft, Writing – review & editing. **Dayna R. Cenin:** Formal analysis, Methodology, Software, Validation, Visualization, Writing – review & editing. **Pei Li:** Validation, Writing – review & editing. **Sha Tao:** Conceptualization, Writing – review & editing. **Chen Yang:** Resources, Validation, Writing – review & editing. **Bei Yan:** Funding acquisition, Project administration, Resources, Validation, Writing – review & editing. **Iris Lansdorp-Vogelaar:** Project administration, Supervision, Validation, Funding acquisition, Writing – review & editing.

## Declaration of Competing Interest

The authors declare that they have no known competing financial interests or personal relationships that could have appeared to influence the work reported in this paper.

## References

[b0005] Sung H., Ferlay J., Siegel R.L., Laversanne M., Soerjomataram I., Jemal A. (2021). Global cancer statistics 2020: GLOBOCAN estimates of incidence and mortality worldwide for 36 cancers in 185 countries. CA Cancer J Clin..

[b0010] Pan R., Zhu M., Yu C., Lv J., Guo Y., Bian Z. (2017). Cancer incidence and mortality: A cohort study in China, 2008–2013. Int. J. Cancer.

[b0015] Schreuders E.H., Ruco A., Rabeneck L., Schoen R.E., Sung J.J.Y., Young G.P. (2015). Colorectal cancer screening: a global overview of existing programmes. Gut.

[b0020] Li X., Qian M., Zhao G., Yang C., Bao P., Chen Y. (2019).

[b0025] Bao P., Zheng Y., Wu C., Huang Z., Gao Y., Jin F. (2016). Cancer incidence in urban Shanghai, 1973–2010: an updated trend and age-period-cohort effects. BMC cancer..

[b0030] Li X, Deng Y, Tang W, Sun Q, Chen Y, Yang C, et al. Urban-Rural Disparity in Cancer Incidence, Mortality, and Survivals in Shanghai, China, During 2002 and 2015. Frontiers in Oncology. 2018;8(579). 10.3389/fonc.2018.00579 PMC6287035 30560091

[b0035] Meng W., Cai S.R., Zhou L., Dong Q., Zheng S., Zhang S.-Z. (2009). Performance value of high risk factors in colorectal cancer screening in China. World J. Gastroenterology: WJG..

[b0040] Gong Y.M., Peng P., Bao P.P., Zhong W., Shi Y., Gu K. (2018). The implementation and first-round results of a community-based colorectal cancer screening program in shanghai. China. Oncologist..

[b0045] Fraser C.G., Allison J.E., Halloran S.P., Young G.P. (2012). On behalf of expert working group on fecal immunochemical tests for hemoglobin CCSCWEO. A proposal to standardize reporting units for fecal immunochemical tests for hemoglobin. J. Natl. Cancer Inst..

[b0050] Li P, Zhu P, Song R, Tao S. Shi Qi Zhong Mian Yi Fa Fen Bian Qian Xue Shi Yan Jian Ce Xing Neng Ping Gu [Performance evaluation of 17 fecal immunochemical tests] Jian Yan Yi Xue [Laboratory Medicine] 2019;34(2):7.

[b0055] Loeve F., Boer R., van Oortmarssen G.J., van Ballegooijen M., Habbema J.D. (1999). The MISCAN-COLON simulation model for the evaluation of colorectal cancer screening. Comput. Biomed. Res..

[b0060] Loeve F, Boer R, van Ballegooijen M, van Oortmarssen G, Habbema J. Final Report MISCAN-COLON microsimulation model for colorectal cancer: report to the National Cancer Institute Project No. NO1-CN55186. Rotterdam; 1998.

[b0065] van Hees F., Zauber A.G., van Veldhuizen H., Heijnen M.L., Penning C., de Koning H.J. (2015). The value of models in informing resource allocation in colorectal cancer screening: the case of The Netherlands. Gut.

[b0070] Gini A., van Ravesteyn N.T., Jansen E.E., Heijnsdijk E.A., Senore C., Anttila A. (2021). The EU-TOPIA evaluation tool: An online modelling-based tool for informing breast, cervical, and colorectal cancer screening decisions in Europe. Prev. Med. Rep..

[b0075] Gong YM, Wu C, Zhang M, Peng P, Gu K, Bao PP, et al. Shanghai Ren Qun Jie Zhi Chang Ai Sheng Cun Lv Fen Xi [Colorectal cancer survival analysis in major areas in Shanghai China]. Zhongguo Ai Zheng Za Zhi [China Oncology]. 2015;25(7):497-504.

[b0080] Shanghai Shi Ji Bing Yu Fang Kong Zhi Zhong Xin [Shanghai Municipal Center for Disease Control and Prevention]. Shanghai Shi She Qu Ju Min Da Chang Ai Shai Cha Di Yi Lun Ping Gu Bao Gao [Evaluation report of the first-round colorectal cancer screening program in Shanghai]. Shanghai: Shanghai Shi Ji Bing Yu Fang Kong Zhi Zhong Xin [Shanghai Municipal Center for Disease Control and Prevention]; 2016.

[b0085] Guo Wu Yuan Ren Kou Pu Cha Ban Gong Shi [Population Census Office under the State Council], Guo Jia Tong Ji Ju Ren Kou He Jiu Ye Tong Ji Si [Department of Population and Employment Statistics National Bureau of Statistics]. Zhongguo 2010 Nian Ren Kou Pu Cha Zi liao [Tabulation of the 2010 population Census of the People's Republic of China]. Table 6-4 Quan Guo Fen Nian Ling Xing Bie De Si Wnag Ren Kou Zhuang Kuang [Nationwide death population by age and sex] (2009.11.1-2010.10.31) [Internet]. Zhongguo Tong Ji Chu Ban She [China Statistics Press]; 2010 [Available from: http://www.stats.gov.cn/english/Statisticaldata/CensusData/rkpc2010/indexce.htm.

[b0090] Hassan C., Quintero E., Dumonceau J.M., Regula J., Brandao C., Chaussade S. (2013). Post-polypectomy colonoscopy surveillance: European Society of Gastrointestinal Endoscopy (ESGE) Guideline. Endoscopy..

[b0095] Gong YM, Gu K, Peng P, Wu CX, Zheng Y. She Qu Ju Min Da Chang Ai Shai Cha Gong Zuo Gui Fan Jie Du [Interpretation of the Guidelines for Screening of Colorectal Cancer in Community Residents] Shanghai Yu Fang Yi Xue [Shanghai Preventive Medicine] 2017;29(2):3.

[b0100] Zhonghua Yi Xue Hui Xiao Hua Nei Jing Xue Fen Hui [Chinese Society of Digestive Endoscopy of the Chinese Medical Association], Zhongguo Kang Ai Xie Hui Zhong Liu Nei Jing Xue Zhuan Ye Wei Yuan Hui [The Society of Tumor Endoscopy of the Chinese Anti-Cancer Association]. Zhongguo Zao Qi Jie Zhi Chang Ai Shai Cha Ji Nei Jing Zhen Zhi Zhi Nan (Beijing, 2014)]. [Chinese guideline on the screening and endoscopic management of early colorectal cancer (Beijing, 2014)]. Wei Chang Bing Xue [Chinese Journal of Gastroenterology]. 2015;20(6):21.

[b0105] Chinese Society of Clinical Oncology (CSCO) diagnosis and treatment guidelines for colorectal cancer working group. Chinese Society of Clinical Oncology (CSCO) diagnosis and treatment guidelines for colorectal cancer 2018 (English version). Chin J Cancer Res. 2019;31(1):117-34. 10.21147/j.issn.1000-9604.2019.01.07 PMC6433585 30996570

[b0110] Endoscopology. DECEDaTGoCSoD, Gastroenterology. DSOGoCSo, Endoscopology. EGoCSoD, Gastroenterology. DPGoCSo (2015). Zhong guo zao qi jie zhi chang ai ji ai qian bing bian shai cha yu zhen zhi gong shi [Consensus on screening and diagnosis of early colorectal cancer and precancerous lesions in China]. Zhong Guo Shi Yong Nei Ke Za Zhi [Chinese Journal of Practical Internal Medicine]..

[b0115] Sung J.J., Ng S.C., Chan F.K., Chiu H.M., Kim H.S., Matsuda T. (2015). An updated Asia Pacific Consensus Recommendations on colorectal cancer screening. Gut.

[b0120] Goede S.L., van Roon A.H., Reijerink J.C., van Vuuren A.J., Lansdorp-Vogelaar I., Habbema J.D. (2013). Cost-effectiveness of one versus two sample faecal immunochemical testing for colorectal cancer screening. Gut.

[b0125] Hol L., van Leerdam M.E., van Ballegooijen M., van Vuuren A.J., van Dekken H., Reijerink J.C. (2010). Screening for colorectal cancer: randomised trial comparing guaiac-based and immunochemical faecal occult blood testing and flexible sigmoidoscopy. Gut.

[b0130] Hol L., Wilschut J.A., van Ballegooijen M., van Vuuren A.J., van der Valk H., Reijerink J.C. (2009). Screening for colorectal cancer: random comparison of guaiac and immunochemical faecal occult blood testing at different cut-off levels. Br. J. Cancer..

[b0135] van Rossum L.G., van Rijn A.F., Laheij R.J., van Oijen M.G., Fockens P., van Krieken H.H. (2008). Random comparison of guaiac and immunochemical fecal occult blood tests for colorectal cancer in a screening population. Gastroenterology.

[b0140] van Roon A.H., Wilschut J.A., Hol L., van Ballegooijen M., Reijerink J.C., t Mannetje H (2011). Diagnostic yield improves with collection of 2 samples in fecal immunochemical test screening without affecting attendance. Clin. Gastroenterol. Hepatol..

[b0145] Rutter C.M., Knudsen A.B., Marsh T.L., Doria-Rose V.P., Johnson E., Pabiniak C. (2016). Validation of models used to inform colorectal cancer screening guidelines: accuracy and implications. Med. Decis. Making.

[b0150] van der Meulen M.P., Lansdorp-Vogelaar I., van Heijningen E.M., Kuipers E.J., van Ballegooijen M. (2016). Nonbleeding adenomas: Evidence of systematic false-negative fecal immunochemical test results and their implications for screening effectiveness-A modeling study. Cancer.

[b0155] van Rijn J.C., Reitsma J.B., Stoker J., Bossuyt P.M., van Deventer S.J., Dekker E. (2006). Polyp miss rate determined by tandem colonoscopy: a systematic review. Am. J. Gastroenterol..

[b0160] Schroy P.C., Coe A., Chen C.A., O'Brien M.J., Heeren T.C. (2013). Prevalence of advanced colorectal neoplasia in white and black patients undergoing screening colonoscopy in a safety-net hospital. Ann. Intern. Med..

[b0165] Wang Z.H., Gao Q.Y., Fang J.Y. (2012). Repeat colonoscopy every 10 years or single colonoscopy for colorectal neoplasm screening in average-risk Chinese: A cost-effectiveness analysis. Asian Pac. J. Cancer Prev..

[b0170] Li X., Wang J., Chen L., Xiang W., Wang D., Wang Z. (2016). Opportunistic screening and mass screening for colorectal neoplasm: A cost-effectiveness analysis. Chinese J. Gastroenterol..

[b0175] Wu Y., Jia H.X., Zhu J. (2014). Da Chang Ai Bing Zhong Zhu Yuan Fei Yong Ying Xiang Yin Su De Yan Jiu [Study on Affecting Factors of Medical Expenses of Colorectal Cancer]. Yi Yao Qian Yan [Medical Frontier]..

[b0180] Inflation Tool. Inflation calculator - Chinese Renminbi [Internet]. Inflation Tool; 2019 [Available from: https://www.inflationtool.com/chinese-renminbi.

[b0185] Lang K., Lines L.M., Lee D.W., Korn J.R., Earle C.C., Menzin J. (2009). Lifetime and treatment-phase costs associated with colorectal cancer: evidence from SEER-Medicare data. Clinical Gastroenterology and Hepatology..

[b0190] Ness R.M., Holmes A.M., Klein R., Dittus R. (1999). Utility valuations for outcome states of colorectal cancer. Am. J. Gastroenterol..

[b0195] Lin G., Feng Z., Liu H., Li Y., Nie Y., Liang Y. (2019). Mass screening for colorectal cancer in a population of two million older adults in Guangzhou. China. Sci. Rep..

[b0200] Wu D., Luo H.Q., Zhou W.X., Qian J.M., Li J.N. (2014). The performance of three-sample qualitative immunochemical fecal test to detect colorectal adenoma and cancer in gastrointestinal outpatients: an observational study. PLoS ONE.

[b0205] Ran T, Cheng C-Y, Misselwitz B, Brenner H, Ubels J, Schlander M. Cost-effectiveness of colorectal cancer screening strategies—a systematic review. Clin. Gastroenterol. Hepatol. 2019;17(10):1969-81. e15. 10.1016/j.cgh.2019.01.014 30659991

[b0210] Cai S.R., Zhu H.H., Huang Y.Q., Li Q.L., Ma X.Y., Zhang S.Z. (2016). Cost-effectiveness between double and single fecal immunochemical test (s) in a mass colorectal cancer screening. Biomed. Res. Int..

[b0215] Wang H., Huang H.Y., Liu C.C., Bai F.Z., Zhu J., Wang L. (2020). Health economic evidence for colorectal cancer screening programs in China: an update from 2009–2018. Zhonghua liu Xing Bing Xue Za Zhi..

[b0220] Liang Q., Li X., Ye G., Hong J., Wang J., Chen L. (2019). Opportunistic screening versus mass screening for colorectal neoplasms in China: a cost-benefit analysis. Int. J. Clin. Exp. Med..

[b0225] Huang W., Liu G., Zhang X., Fu W., Zheng S., Wu Q. (2014). Cost-effectiveness of colorectal cancer screening protocols in populations. PLoS ONE.

[b0230] Allison J.E., Fraser C.G., Halloran S.P., Young G.P. (2014). Population screening for colorectal cancer means getting FIT: the past, present, and future of colorectal cancer screening using the fecal immunochemical test for hemoglobin (FIT). Gut Liver..

[b0235] Huang J.L.W., Chen P., Yuan X., Wu Y., Wang H.H.X., Jiang J.Y. (2016). Tailoring choice between colonoscopy versus sigmoidoscopy for population-based colorectal cancer screening in Chinese patients: a prospective colonoscopy study. The Lancet..

[b0240] Arrospide A., Idigoras I., Mar J., de Koning H., van der Meulen M., Soto-Gordoa M. (2018). Cost-effectiveness and budget impact analyses of a colorectal cancer screening programme in a high adenoma prevalence scenario using MISCAN-Colon microsimulation model. BMC Cancer..

[b9000] Cenin D., Li P., Wang J., de Jonge L., Yan B., Tao S., Lansdorp-Vogelaar I. (2022). Optimising colorectal cancer screening in Shanghai, China: a modelling study. BMJ Open.

[b0245] Yang Y., Huang J., Wu W., Luu H.N., Moy F.M., Tan S. (2020). Performance of initial screening tests for colorectal cancer and subsequent adherence to colonoscopy. An Ecological Study..

[b0250] Cheng S.Y., Li M.C., Chia S.L., Huang K.C., Chiu T.Y., Chan D.C. (2018). Factors affecting compliance with confirmatory colonoscopy after a positive fecal immunochemical test in a national colorectal screening program. Cancer.

[b0255] Wu W., Wang Y., Jiang H., Yang C., Li X., Yan B. (2019). Colorectal cancer screening modalities in Chinese population: Practice and lessons in Pudong New Area of Shanghai. China. Frontiers in Oncology..

[b0260] Sekiguchi M., Igarashi A., Sakamoto T., Saito Y., Esaki M., Matsuda T. (2020).

[b0265] Wong M.C.S., Lam T.Y.T., Tsoi K.K.F., Hirai H.W., Chan V.C.W., Ching J.Y.L. (2014). A validated tool to predict colorectal neoplasia and inform screening choice for asymptomatic subjects. Gut.

[b0270] Symonds E.L., Fraser R.J., Young G.P. (2018). FIT for purpose: enhanced applications for faecal immunochemical tests. J. Lab Precis. Med..

[b0275] Nelder J.A., Mead R. (1965). A simplex method for function minimization. The Computer Journal..

[b0280] Lansdorp-Vogelaar I., van Ballegooijen M., Boer R., Zauber A., Habbema J.D. (2009). A novel hypothesis on the sensitivity of the fecal occult blood test: Results of a joint analysis of 3 randomized controlled trials. Cancer.

[b0285] Shi X., Shan Y., Yu E., Fu C., Meng R., Zhang W. (2014). Lower rate of colonoscopic perforation: 110,785 patients of colonoscopy performed by colorectal surgeons in a large teaching hospital in China. Surg. Endosc..

[b0290] Huang Q.C., Ye D., Jiang X.Y., Li Q.L., Yao K.Y., Wang J.B. (2017). Cost-effectiveness analysis on colorectal cancer screening program. China J. Epidemiol..

