# Supplementary material for: Cost-effectiveness analysis of colorectal cancer screening in Shanghai, China: A modelling study
Source: Prev Med Rep. 2022 Jul 4;29:101891. doi: 10.1016/j.pmedr.2022.101891 (PMC9294625; doi:10.1016/j.pmedr.2022.101891)
Supplement: Supplementary data 1 [file mmc1.docx]

Supplementary Methods

[MISCAN-Colon Model Description 2](#_Toc72765603)

[Model Overview 2](#_Toc72765604)

[Model Outputs 12](#_Toc72765605)

[Tables 14](#_Toc72765606)

[Table S1: Raw data provided by Pudong CDC; Screening invitiations and participations for period 2013-2015 14](#_Toc72765607)

[Table S2: International utility losses associated with colorectal cancer screening and 16](#_Toc72765608)

[Figures 17](#_Toc72765609)

[Figure S7: Screening pathway as reported by Gong(25)and surveillance pathway as reported in Chinese clinical practice guidelines(26). 17](#_Toc72765610)

[Detailed information on the Shanghai CRC screening program 18](#_Toc72765611)

[Test characteristics 19](#_Toc72765612)

[References 20](#_Toc72765613)

## MISCAN-Colon Model Description

### Model Overview

The Microsimulation Screening Analysis-Colon (MISCAN-Colon) model is a stochastic, semi-Markov, microsimulation model that is useful in explaining and predicting trends in CRC incidence and mortality and to quantify the effects and costs of primary prevention of CRC, screening for CRC and surveillance.

The term ‘microsimulation’ implies that individuals are moved through the model one at a time (i.e. as individuals), rather than as proportions of a cohort. This allows future state transitions to depend on past transitions, giving the model a ‘memory’. Furthermore, unlike most traditional Markov models, MISCAN-Colon does not use yearly transition probabilities; instead it generates durations in states, thereby increasing model flexibility and computational performance. The term ‘stochastic’ implies that the model simulates sequences of events by drawing from distributions of probabilities/durations, rather than using fixed values. Hence, the results of the model are subject to random variation. Possible events are birth and death of a person, adenoma incidence and transitions from one state of disease to another.

At two expert meetings at the National Cancer Institute (Bethesda, Maryland, United States of America) held on June 5–7, 1996, and May 12–13, 1997, the structure of the model was devised in agreement with the currently accepted model of the adenoma–carcinoma sequence (Figure 1). MISCAN-Colon consists of 3 modules: a demography module, natural history module, and screening module (Figure 1). Although these parts are not physically separated in MISCAN-Colon, it is useful to consider them separately.


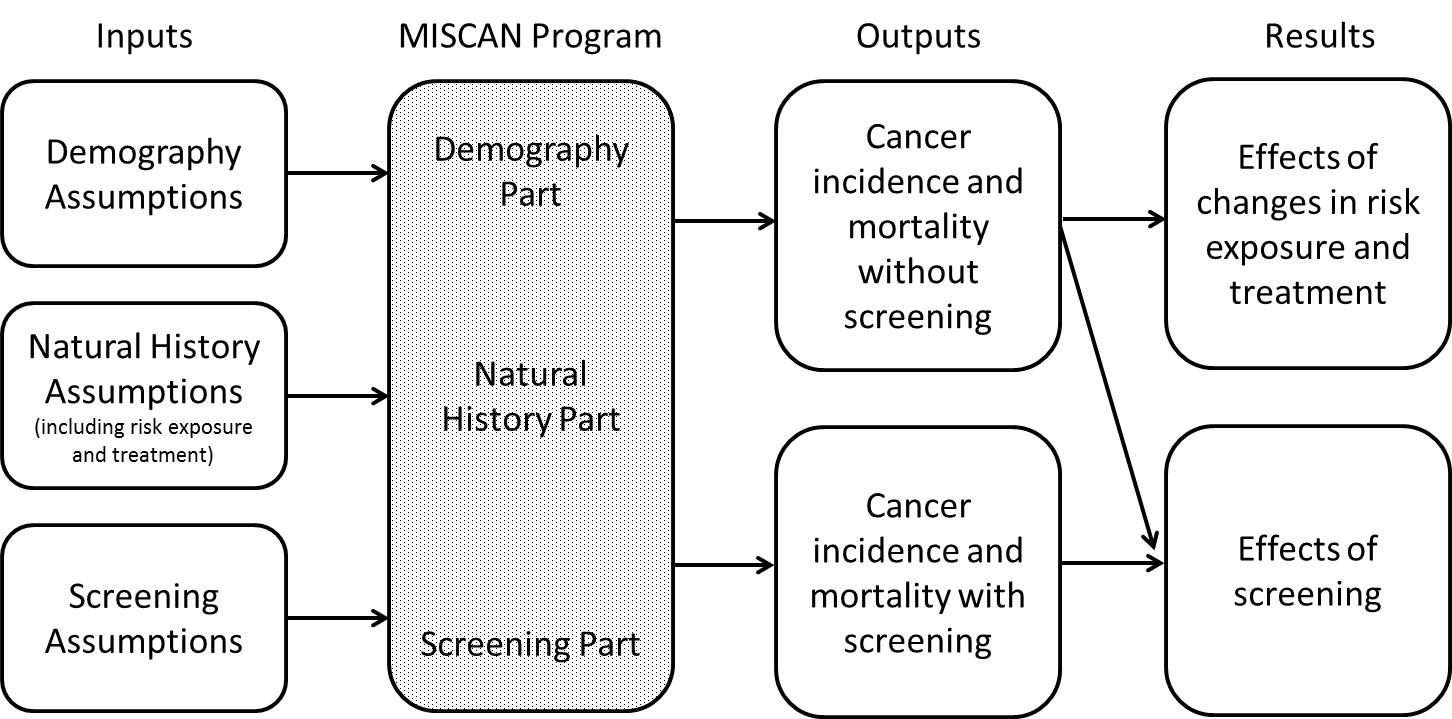


Figure S1: Structure of MISCAN-Colon

#### Demography module

The demography module of MISCAN-Colon simulates individual life histories without colorectal cancer (CRC) to form a population. Using birth tables and life tables representative of the population under consideration, the model draws a date of birth and a date of non-CRC death for each simulated individual. The model restricts the maximum age a person can achieve to 100 years.

#### Natural history module

In the natural history module, MISCAN-Colon simulates the development of CRC in the population. It was assumed that all CRCs are preceded by adenomas. As each simulated individual ages, one or more adenomas may develop (Figure 2). These adenomas can be either progressive or non-progressive and both can grow in size from small (≤5 mm), to medium (6–9 mm), to large (≥10 mm). Only progressive adenomas can develop into preclinical cancer and these may progress through stages I to IV. In every stage there is a chance of the cancer being diagnosed because of symptoms. After clinical diagnosis, CRC survival is simulated using age-, stage-, and localisation-specific survival estimates for clinically diagnosed CRC obtained from a study by Rutter and colleagues.(1) For individuals with synchronous CRCs at time of diagnosis, the survival of the most advanced cancer is used. The date of death for individuals with CRC is set to the earliest simulated death either because of CRC or because of another causes (Demography model).

The average duration between onset of a progressive adenoma and the transition to preclinical cancer was estimated based on the interval cancer rate after a once-only sigmoidoscopy in a randomized controlled trial from the United Kingdom.(2) The duration of cancer in preclinical stages was estimated based on the results of three large randomised controlled screening trails.(3) This resulted in the average duration of 2.5 years, 2.5 years, 3.7 years, and 1.5 years, for stages I-IV respectively, with a total average duration of 6.7 years because not every cancer reaches stage IV before clinical diagnosis. All durations were governed by an exponential probability distribution. Durations in each of the invasive cancer stages as well as durations in the stages of the non-invasive adenomas were assumed to be 100% associated with each other, but the durations in invasive stages as a whole were independent of durations in non-invasive adenoma stages that precede cancer. These assumptions resulted in an exponential distribution of the total duration of progressive non-invasive adenomas and of the total duration of preclinical cancer, which has also been used in other cancer screening models.(4, 5)


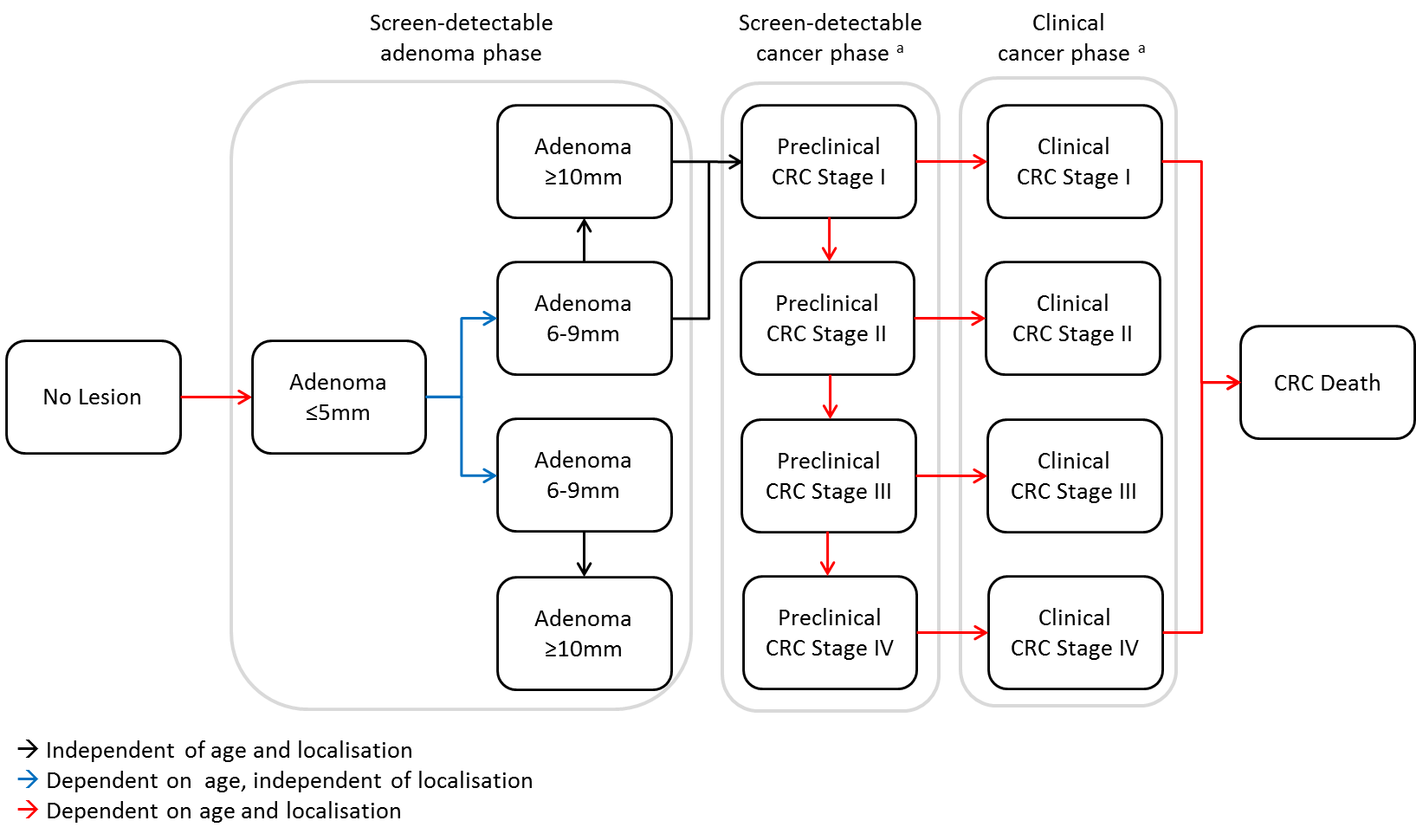


Figure S2: Schematic representation of the natural history module of the MISCAN-Colon model.

CRC: colorectal cancer

The arrows between the states show which types of transitions can occur. In every state before death, a transition to “death from other causes” can occur (state and connect arrows not shown).

1. Cancer stages correspond to the American Joint Committee on Cancer / International Union Against Cancer staging system for CRC.

Based on expert opinion, it is assumed that 30% of the cancers arise from adenomas of 6–9 mm and that 70% arise from larger adenomas. The preclinical incidence of non-progressive adenomas that will never grow into cancer was varied until the simulated prevalence of all adenomas matched with data from autopsy studies.(6-15) The size distribution of adenomas over all ages was assumed to be 73% for stages less than or equal to 5 mm, 15% for stages 6–9 mm, and 12% for stages greater than or equal to 10 mm.(16)

An individual’s risk of developing adenomas depends on the individual’s age and a personal risk index. As a result most individuals will not develop adenomas, whilst others develop many. The distribution of adenomas over the colon and rectum is assumed to equal the distribution of cancers observed before the introduction of screening. The age-specific onset of adenomas and the personal risk index were calibrated to data on the prevalence and multiplicity distribution of adenomas as observed in autopsy studies (Figure 3).(6-15) The age-specific probability of adenoma-progressivity and the age- and localization-specific transition probabilities between preclinical cancer stages and between preclinical and clinical cancer stages were simultaneously calibrated to SEER data on the age-, stage-, and localization-specific incidence of CRC as observed before the introduction of screening (Figure 4).


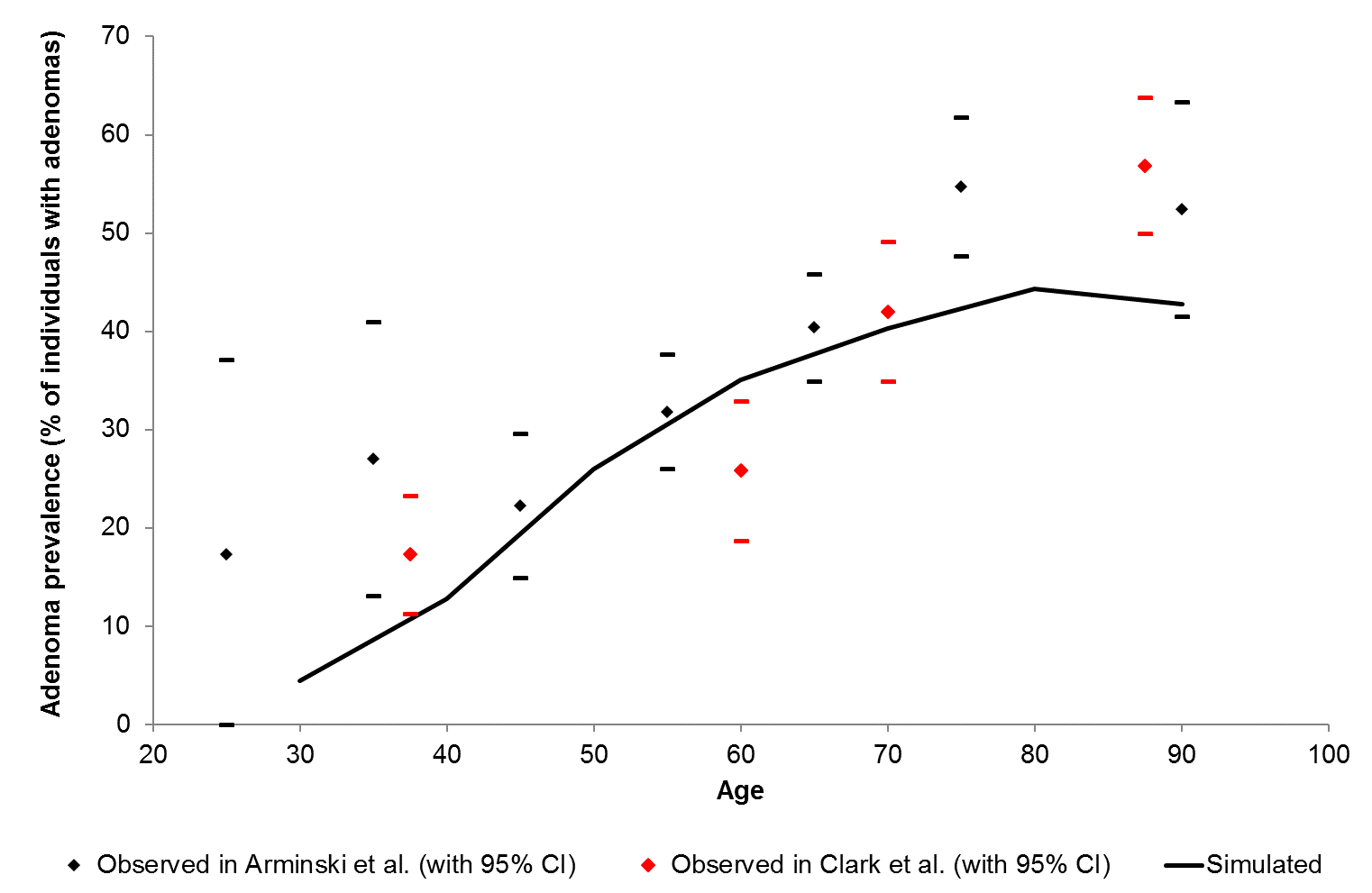


Figure S3: Adenoma prevalence observed in selected autopsy studies vs simulated by MISCAN-Colon (% of individuals with adenomas).*

*Observed results are shown only for the 2 largest studies on which the model has been calibrated.(6, 10) The model has additionally been calibrated to eight other autopsy studies.(7-9, 11-15)

**
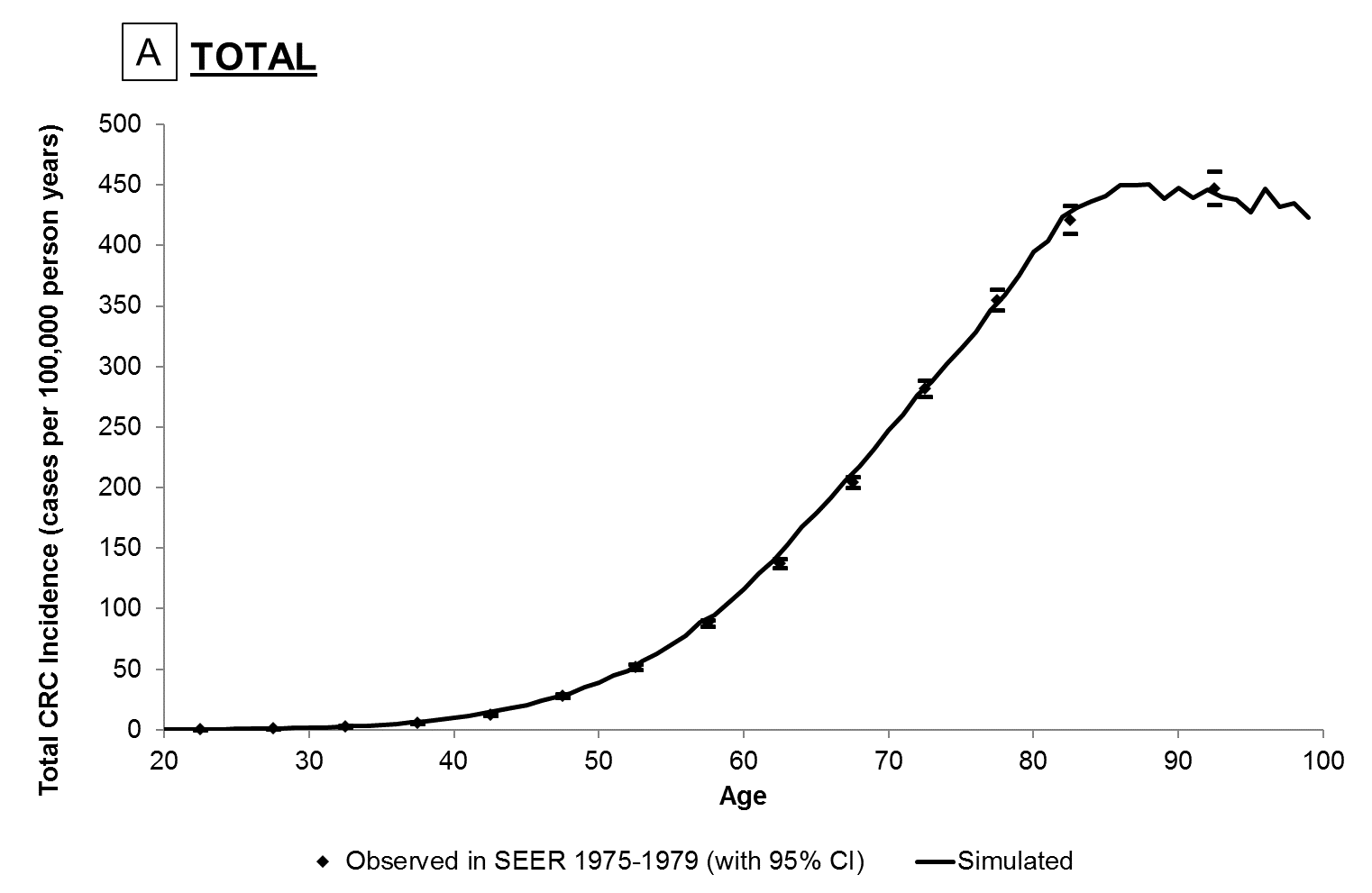
**

**
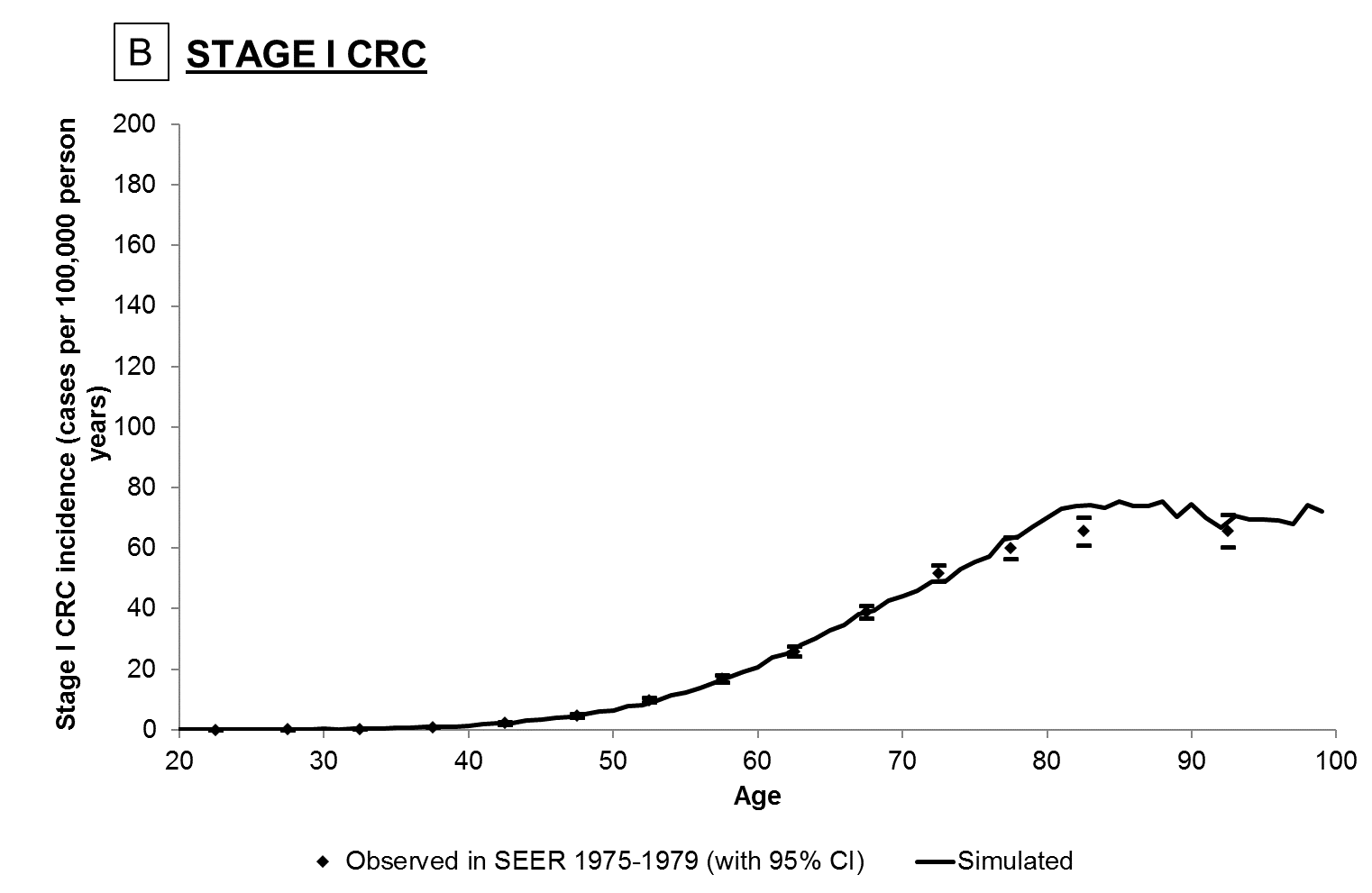
**
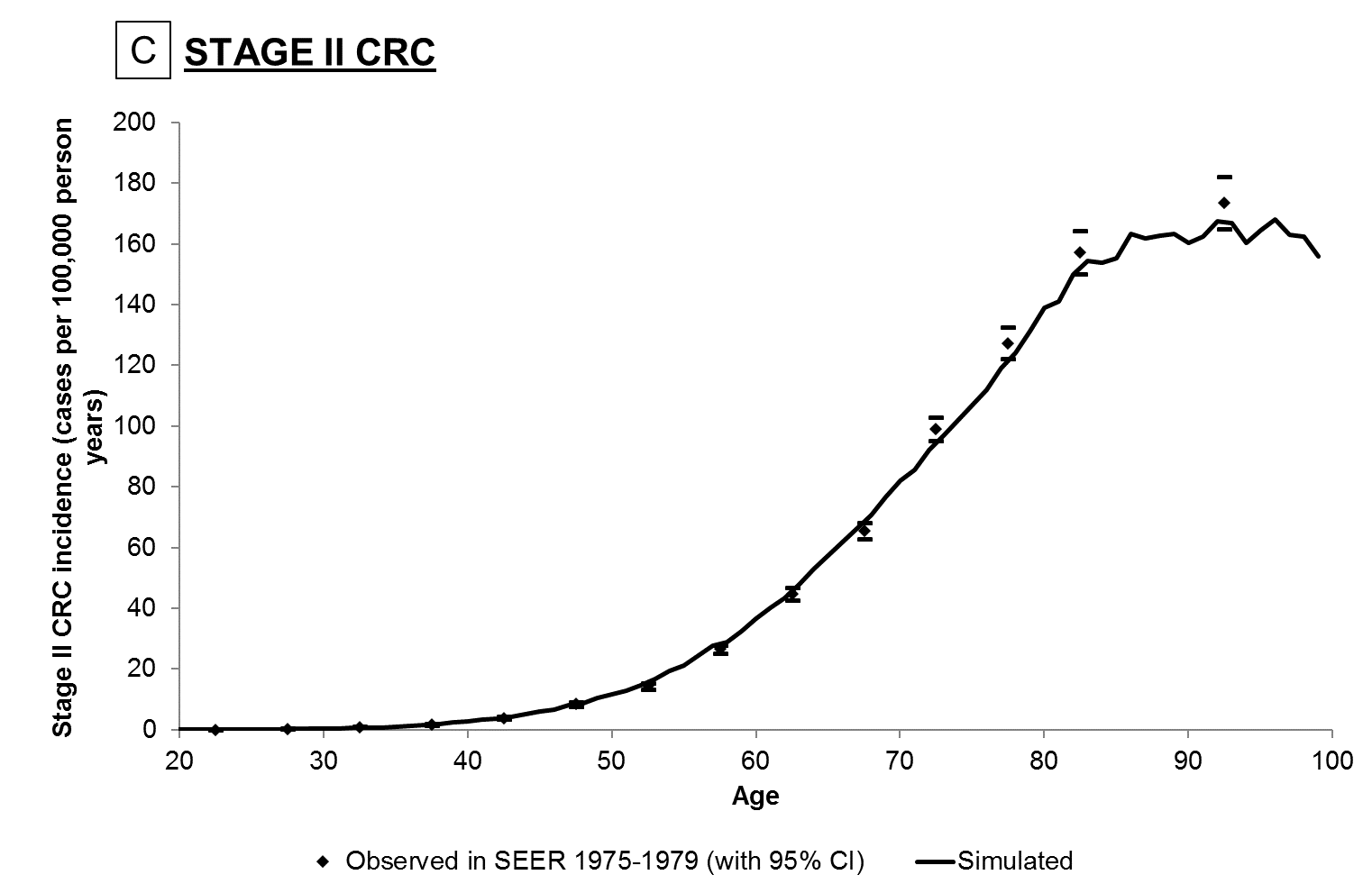


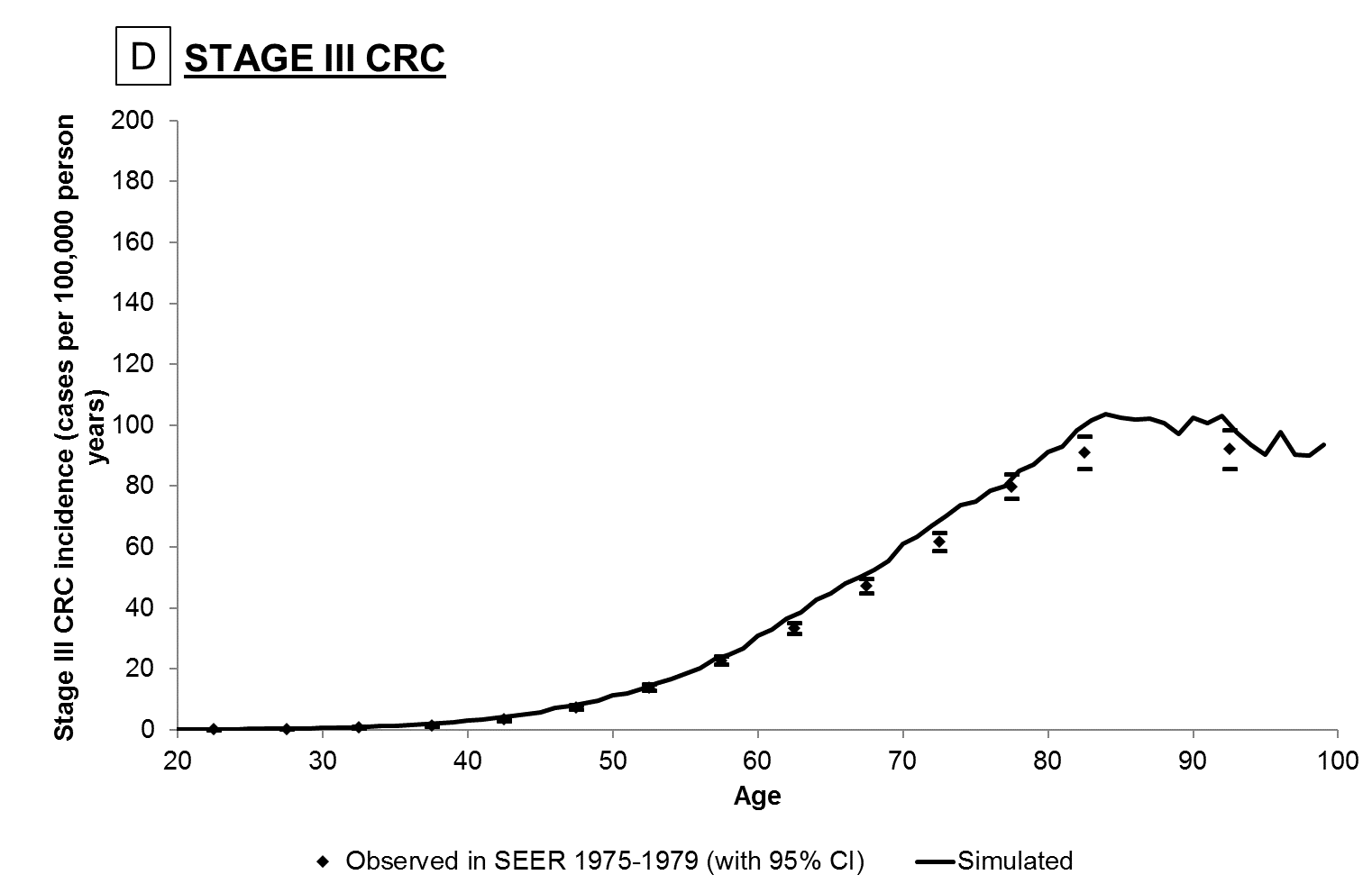

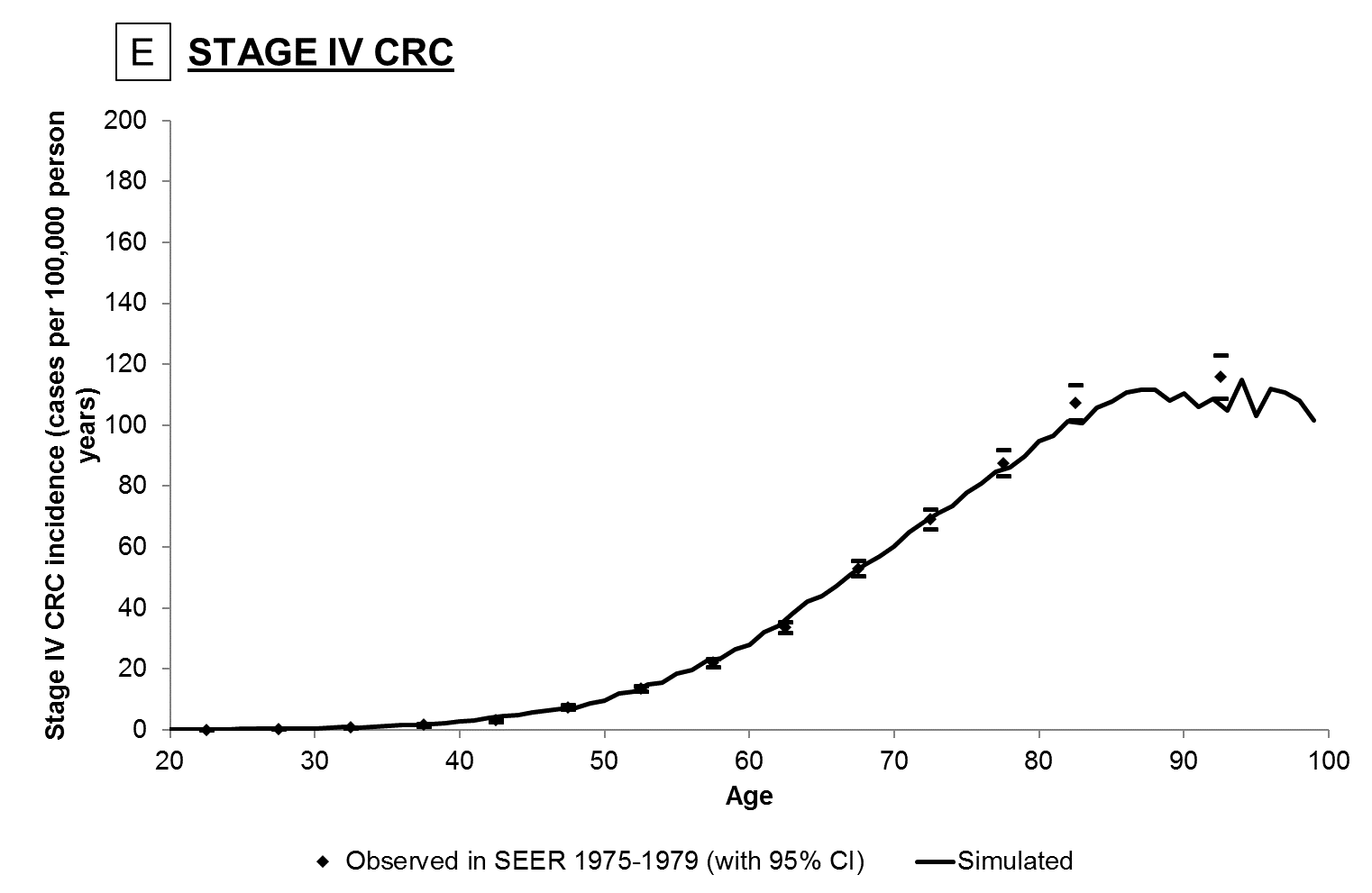


**Figure S4: CRC incidence observed before the introduction of screening vs simulated by MISCAN-Colon (total (A), stage I CRC (B), stage II CRC (C), stage III CRC (D), stage IV CRC (E); cases per 100,000 person years).**

The average durations of the preclinical cancer stages were calibrated to the rates of screen-detected and interval cancers observed in randomized controlled trials evaluating screening using guaiac faecal occult blood tests.(17-19) This exercise has been described extensively in a publication by Lansdorp-Vogelaar and colleagues.(3) The average duration from the emergence of an adenoma until progression into preclinical cancer (i.e. the adenoma dwell-time) was calibrated to the rates of interval cancers (including surveillance detected cancers) observed in a randomized controlled trial evaluating once-only sigmoidoscopy screening (Figure 5).(2)

Furthermore, we assume: i) an equal overall dwell-time for adenomas developing into CRC from a medium size (30% of all CRCs) and from a large size (70% of all CRCs); exponential distribution for all durations in the adenoma and preclinical cancer phase; perfect correlation for the duration in the adenoma and preclinical cancer (meaning that if a small adenoma progresses rapidly to a medium-sized adenoma, it will also progress rapidly to a large adenoma or to a preclinical cancer stage I); and absence of correlation between durations in the adenoma phase and duration in the preclinical cancer phase.

The stage-specific survival of patients with screen-detected cancer is assumed to be the same as the survival of patients with cancers clinically diagnosed in the same stage, except if screen-detection occurs in the same stage as the cancer would have been diagnosed without screening.(20) In that case, survival is assumed to be similar to survival of one stage more favourable (i.e. stage II cancer gets stage I survival). Only if screen-detected in stage IV, we assume no possibility for within-stage shift and stage IV screen detected cancers always have the same survival as clinically diagnosed cancers in stage IV. Removal of an adenoma always prevents development of any subsequent cancer that may have arisen from this adenoma.


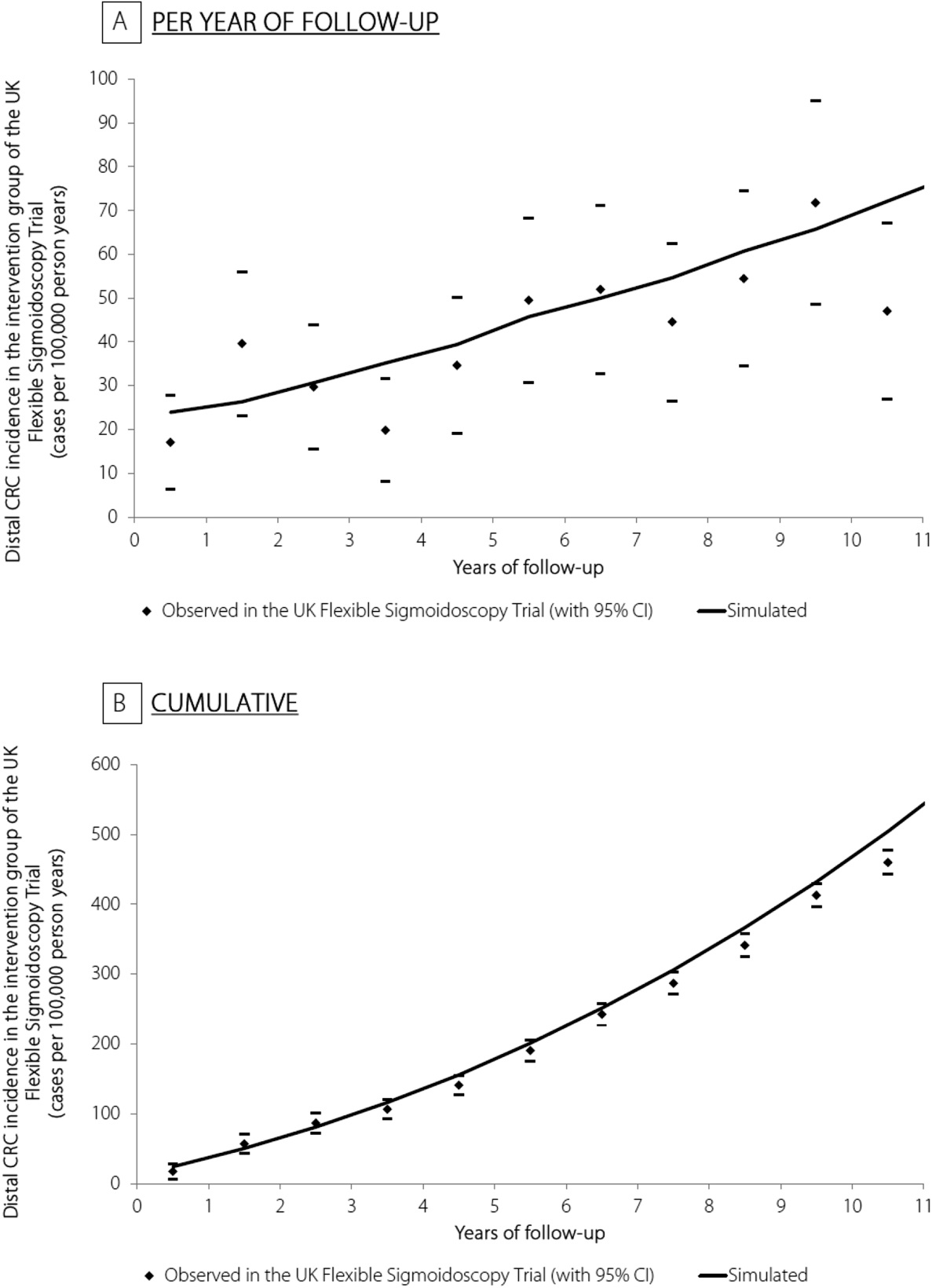


Figure S5: Distal CRC incidence observed in the intervention group of the UK Flexible Sigmoidoscopy Trial vs simulated by MISCAN-Colon (per year of follow-up (A), cumulative (B); cases per 100,000 person years).

#### Screening module

Screening interrupts the development of CRC and therefore alters some of the simulated life histories. With screening, some cancers will be prevented by the detection and removal of adenomas; other cancers will be detected in an earlier stage than with clinical diagnosis which offers a more favourable survival. In this way, screening prevents CRC incidence or CRC death. The life-years gained by screening are calculated by comparing the model-predicted life-years lived in the population with and without screening. The effects of different screening policies can be compared by applying them to identical natural histories. As seen in RCTs on guaiac faecal occult blood testing, the stage-specific survival of screen-detected CRC was more favourable compared with clinically detected CRC, even after the lead-time bias correction.(3) We therefore assign screen-detected cancers that would have been clinically detected in the same stage the survival corresponding to a cancer that is one stage less progressive. For example, a cancer which is screen-detected in stage II, that would also have been clinically diagnosed in stage II, is assigned the survival of a clinically diagnosed stage I cancer. The only exceptions were screen-detected stage IV cancers. These cancers were always assigned the survival of a clinically diagnosed stage IV cancer.

In addition to modelling positive health effects of screening, we also model colonoscopy-related complications, over-diagnosis and over-treatment of CRC (ie, the detection and treatment of cancers that would not have been diagnosed without screening).(21-23)

#### Integration of the model components

For each individual, the demography module of MISCAN-Colon simulates a date of birth and a date of death of other causes than CRC, creating a life history without adenomas or CRC.

In patient A in Figure 6, the natural history module generates an adenoma. This adenoma progresses into preclinical cancer (diagnosed as stage II CRC because of symptoms) and results in CRC death before non-CRC death would have occurred. However, in the screening module, a screening examination is introduced (indicated by the blue arrow). During this examination, the adenoma is detected and then removed, and both CRC and CRC death prevented. Hence, in Patient A, the positive effect of the screening intervention is indicated by the green arrow and represents the increased LYG for this patient because of screening.

Patient B also develops an adenoma, and although this adenoma does progress into preclinical cancer, Patient B would never have been diagnosed with CRC in a scenario without screening (see life history 2). However, during the simulated screening examination (blue arrow) CRC is screen-detected in stage I and for this patient, the screening results in over-diagnosis and overtreatment of CRC: in this situation, screening does not prolong life, but it does result in additional LYs with CRC care (over-treatment) as indicated by the red arrow.


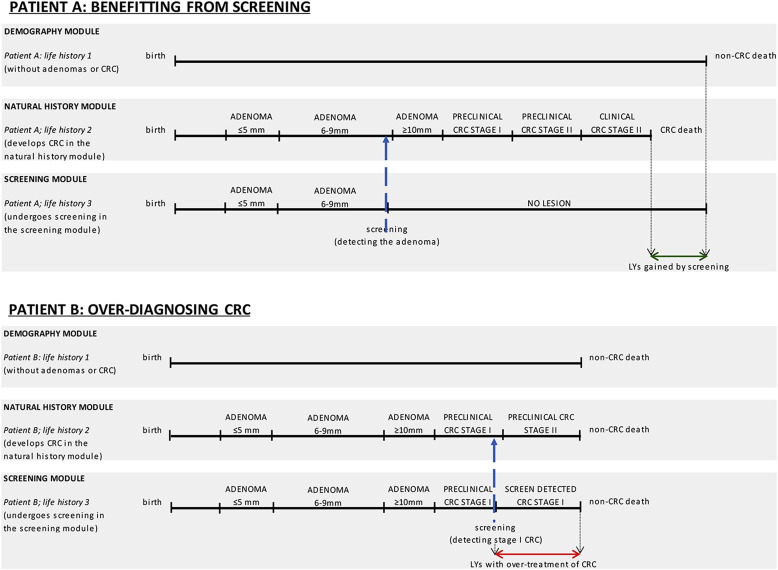


Figure S6: Integrating modules: two example individuals (A and B).

### Model Outputs

The model generates the following output, both undiscounted and discounted:

#### Demography

1. Life-years lived in the population by calendar year and age
2. Deaths from other causes than CRC by calendar year and age

#### Natural history

1. CRC cases by calendar year, stage and age
2. CRC deaths by calendar year and age
3. Life-years lived with CRC by calendar year, stage and age
4. Total number of life years with surveillance for adenoma patients
5. Total number of life years with initial therapy after screen-detected or clinical invasive cancer by stage
6. Total number of life years with continuing therapy after screen-detected or clinical invasive cancer by stage
7. Total number of life years with terminal care before death from other causes by stage
8. Total number of life years with terminal care before death from CRC by stage

#### Screening

1. Number of invitations for screen-tests, screen-tests, diagnostic tests, surveillance and opportunistic screen tests by calendar year
2. Number of positive and negative test results per preclinical state and per year
3. Total number of life years lived, life years lost due to cancer, number of specific deaths and non-specific deaths
4. Number of screenings that prevented cancer by year of screening
5. Number of screenings that detected cancer early by year of screening
6. Number of surveillance tests that prevented cancer by year of surveillance
7. Number of surveillance tests that detected cancer early by year of surveillance
8. Number of life years gained due to screening by year of screening

## **Tables**

### Table S1: Raw data provided by Pudong CDC; Screening invitiations and participations for period 2013-2015

|  | **Individuals invited to screen  (target population)** | **Number of participants** | **Participation Rate** | **Number of Negative Tests** | **Number either positive** | **Number of FIT positive** | **Number of RA positive** | **Both Positive** |
| --- | --- | --- | --- | --- | --- | --- | --- | --- |
| **40-44** | 190,643 | 291 | 0.15% | 254 | 37 | 21 | 18 | 2 |
| **45-49** | 192,640 | 4,214 | 2.19% | 3,667 | 547 | 314 | 271 | 38 |
| **50-54** | 250,887 | 31,574 | 12.58% | 26,489 | 5,085 | 2,859 | 2,569 | 343 |
| **55-59** | 297,673 | 73,692 | 24.76% | 60,026 | 13,666 | 8,137 | 6,595 | 1,066 |
| **60-64** | 260,822 | 104,482 | 40.06% | 82,615 | 21,867 | 13,329 | 10,387 | 1,849 |
| **65-69** | 174,253 | 107,527 | 61.71% | 83,631 | 23,896 | 14,931 | 11,167 | 2,202 |
| **70-74** | 100,008 | 52,834 | 52.83% | 40,295 | 12,539 | 7,690 | 6,060 | 1,211 |
| **75-79** | 89,034 | 29,754 | 33.42% | 22,279 | 7,475 | 4,780 | 3,550 | 855 |
| **80-** | 131,018 | 24,303 | 18.55% | 18,438 | 5,865 | 3,933 | 2,572 | 640 |
| **Total** | 1,686,978 | 428,671 | 25.41% | 337,694 | 90,977 | 55,994 | 43,189 | 8,206 |
|  |  |  |  |  |  |  |  |  |
| Of those who were FIT positive | | | | | | | | |
|  | **Individuals with colonscopy** | **Participation rate** | **No lesion detected** | **Adenomas** | **Advanced adenomas** | **Colorectal cancers** | **others** | **enteritis** |
| **40-44** | 6 | 28.57% | 4 | 2 | 0 | 0 |  |  |
| **45-49** | 105 | 33.44% | 75 | 12 | 9 | 2 | 4 | 3 |
| **50-54** | 947 | 33.12% | 702 | 128 | 73 | 4 | 13 | 27 |
| **55-59** | 2,914 | 35.81% | 1,987 | 449 | 298 | 37 | 65 | 78 |
| **60-64** | 4,704 | 35.29% | 2,987 | 841 | 563 | 101 | 67 | 145 |
| **65-69** | 4,689 | 31.40% | 2,898 | 846 | 622 | 105 | 102 | 116 |
| **70-74** | 2,193 | 28.52% | 1,317 | 394 | 308 | 72 | 53 | 49 |
| **75-79** | 924 | 19.33% | 500 | 175 | 137 | 63 | 28 | 21 |
| **80-** | 433 | 11.01% | 273 | 70 | 29 | 39 | 15 | 7 |
| **Total** | 16,915 | 30.21% | 10,743 | 2,917 | 2,039 | 423 | 347 | 446 |
|  |  |  |  |  |  |  |  |  |
| Of those who were either FIT or RA positive | | | | | | | | |
|  | **Individuals with colonscopy** | **Participation rate** | **No lesion detected** | **Adenomas** | **Advanced adenomas** | **Colorectal cancers** | **others** | **enteritis** |
| **40-44** | 10 | 27.03% | 8 | 2 | 0 | 0 | 0 | 0 |
| **45-49** | 145 | 26.51% | 106 | 17 | 12 | 2 | 5 | 3 |
| **50-54** | 1,236 | 24.31% | 924 | 162 | 91 | 4 | 16 | 39 |
| **55-59** | 3,679 | 26.92% | 2,570 | 559 | 333 | 38 | 74 | 105 |
| **60-64** | 5,967 | 27.29% | 3,941 | 1,011 | 644 | 105 | 81 | 185 |
| **65-69** | 5,739 | 24.02% | 3,653 | 986 | 733 | 107 | 116 | 144 |
| **70-74** | 2,719 | 21.68% | 1,692 | 472 | 354 | 76 | 61 | 64 |
| **75-79** | 1,108 | 14.82% | 629 | 206 | 151 | 65 | 32 | 25 |
| **80-** | 483 | 8.24% | 305 | 77 | 35 | 40 | 16 | 10 |
| **Total** | 21,086 | 23.18% | 13,828 | 3,492 | 2,353 | 437 | 401 | 575 |

Abbreviations: FIT, faecal immunochemical test; RA, risk assessment

Table S2: International utility losses associated with colorectal cancer screening and treatment

| **UTILITY LOSS (QALYs) ^a^** | | | | |
| --- | --- | --- | --- | --- |
| Per FIT |  |  |  | 0 |
| Per colonoscopy ^b^ |  |  |  | 0.00274 |
| Per perforation during colonoscopy ^c^ |  |  |  | 0.00548 |
| Per LY with CRC Care ^d,e^ | Initial Care | Continuing Care | Terminal care  (Death CRC) | Terminal care (Death OC) |
| Stage I | 0.12 | 0.05 | 0.70 | 0.05 |
| Stage II | 0.18 | 0.05 | 0.70 | 0.05 |
| Stage III | 0.24 | 0.24 | 0.70 | 0.24 |
| Stage IV | 0.70 | 0.70 | 0.70 | 0.70 |

Abbreviations: CRC, Colorectal Cancer; FIT, faecal immunochemical test; OC, Other Cause; QALY, Quality-Adjusted Life Year; LY, Life Year

1. The loss of quality of life associated with a particular event.
2. Equal to 2 days per colonoscopy at a utility of 0.5.
3. Perforations associated with colonoscopy were assumed to be equal to 4 days at a utility of 0.5.
4. Care for CRC was divided in three clinically relevant phases: the initial, continuing, and terminal care phase. The initial care phase was defined as the first 12 months after diagnosis; the terminal care phase was defined as the final 12 months of life; the continuing care phase was defined as all months in between. In the terminal care phase, we distinguished between CRC patients dying from CRC and CRC patients dying from another cause. For patients surviving less than 24 months, the final 12 months were allocated to the terminal care phase and the remaining months were allocated to the initial care phase.
5. Utility losses for LYs with initial care were derived from a study by Ness and colleagues. (24) For LYs with continuing care for stage I and II CRC, we assumed a utility loss of 0.05 QALYs; for LYs with continuing care for stage III and IV CRC, we assumed the corresponding utility losses for LYs with initial care. For LYs with terminal care for CRC, we assumed the utility loss for LYs with initial care for stage IV CRC. For LYs with terminal care for another cause, we assumed the corresponding utility losses for LYs with continuing care.

## Figures


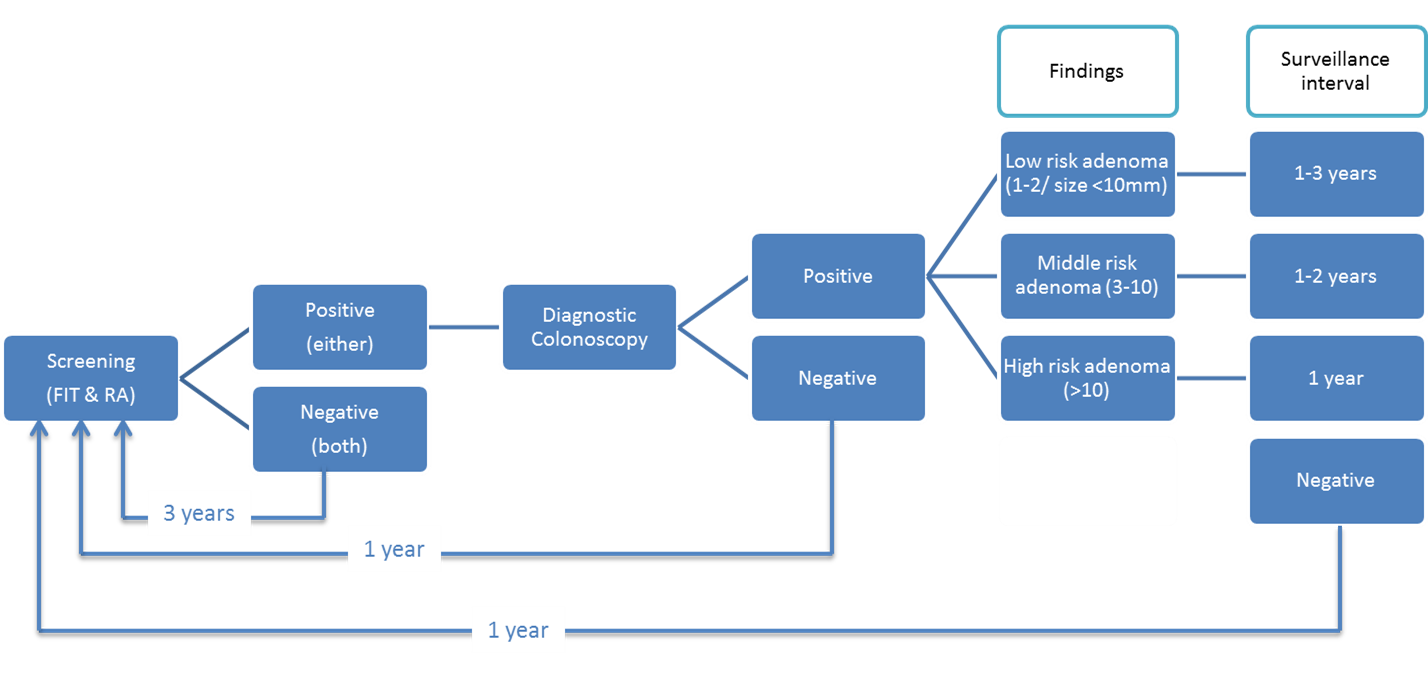


### Figure S7: Screening pathway as reported by Gong(25)and surveillance pathway as reported in Chinese clinical practice guidelines(26).

Note: the used surveillance interval after finding a low risk adenoma is 3 years and after finding a middle risk adenoma 2 years.

Figure S8: CRC incidence observed before the introduction of screening vs simulated by MISCAN-Colon in Shanghai; cases per 100,000 person years).

## Detailed information on the Shanghai CRC screening program

The Shanghai FIT consists of two faecal samples taken within a 7 day period. Each sample should be returned to a community health centre within 48 hours. If either one or both of the FITs was positive, a participant was identified as FIT-positive. The RA was conducted either before or after performing the Shanghai FIT. The RA questionnaire consisted of nine questions about risk factors. A positive RA means: (i) participants had one of the following events: (a) a history of cancer, (b) a history of polyps, or (c) a family history of CRC in a first-degree relative; and/or (ii) At least 2 of the following events: (a) chronic constipation (constipation has been longer than two months per year in the past two years); (b) chronic diarrhoea (diarrhoea has lasted more than 3 months cumulatively in the past two years, and the duration of each episode is 1 week above); (c) mucoid bloody faeces; (d) psychological trauma or serious unhappy life events; (e) chronic appendicitis or appendectomy; or (f) chronic cholecystitis or cholecystectomy.(26)

## Test characteristics

In all instances, the estimated positivity and detection rates matched the observed positivity and detection rates within 0.1% (Table 1). Moreover, the calibrated positivity and detection rates were within the 95% confidence interval. For example, the observed positivity rates for the Shanghai FIT and the Shanghai FIT+RA according to the data provided by Pudong CDC was 0.145 and 0.231, respectively. These results were matched in the estimation.

All sensitivities of the Shanghai FIT+RA were higher compared to the sensitivities of the Shanghai FIT alone (Table 2). Comparing these results with the validated FIT, the sensitivities for the Shanghai FIT+RA were higher except for the sensitivity for large adenomas (46.9% compared to 33.0%). The Shanghai FIT showed lower sensitivities compared to the validated FIT except for the sensitivity for medium adenomas (8.7% and 7.1% respectively). In contrast to the sensitivities, the specificity of the Shanghai FIT+RA was much lower (79.3%) compared to the specificity of the validated FIT (96.7%) and the Shanghai FIT (87.4%).

## References

1. Rutter CM, Knudsen AB, Marsh TL, Doria-Rose VP, Johnson E, Pabiniak C, et al. Validation of Models Used to Inform Colorectal Cancer Screening Guidelines: Accuracy and Implications. Med Decis Making. 2016; 36(5): 604-614. [https://doi.org/10.1177/0272989X15622642](https://doi.org/10.1177%2F0272989X15622642)

2. Atkin WS, Edwards R, Kralj-Hans I, Wooldrage K, Hart AR, Northover JM, et al. Once-only flexible sigmoidoscopy screening in prevention of colorectal cancer: a multicentre randomised controlled trial. Lancet. 2010;375(9726):1624-33.

3. Lansdorp-Vogelaar I, van Ballegooijen M, Boer R, et al. A novel hypothesis on the sensitivity of the fecal occult blood test: Results of a joint analysis of 3 randomized controlled trials. Cancer. 2009;115(11):2410-2419. <https://doi.org/10.1002/cncr.24256>

4. Gyrd-Hansen D, Sogaard J, Kronborg O. Analysis of screening data: colorectal cancer. Int J Epidemiol. 1997;26(6):1172-81.

5. Launoy G, Smith TC, Duffy SW, Bouvier V. Colorectal cancer mass-screening: estimation of faecal occult blood test sensitivity, taking into account cancer mean sojourn time. Int J Cancer. 1997;73(2):220-4.

6. Arminski TC, McLean DW. Incidence and Distribution of Adenomatous Polyps of the Colon and Rectum Based on 1,000 Autopsy Examinations. Dis Colon Rectum. 1964;7:249-61.

7. Blatt L. Polyps of the colon and rectum. Dis Colon Rectum. 1961;4:277-82.

8. Bombi JA. Polyps of the colon in Barcelona, Spain. An autopsy study. Cancer. 1988;61(7):1472-6.

9. Chapman I. Adenomatous polypi of large intestine: incidence and distribution. Ann Surg. 1963;157:223-6.

10. Clark JC, Collan Y, Eide TJ, Esteve J, Ewen S, Gibbs NM, et al. Prevalence of polyps in an autopsy series from areas with varying incidence of large-bowel cancer. Int J Cancer. 1985;36(2):179-86.

11. Jass JR, Young PJ, Robinson EM. Predictors of presence, multiplicity, size and dysplasia of colorectal adenomas. A necropsy study in New Zealand. Gut. 1992;33(11):1508-14.

12. Johannsen LG, Momsen O, Jacobsen NO. Polyps of the large intestine in Aarhus, Denmark. An autopsy study. Scand J Gastroenterol. 1989;24(7):799-806.

13. Rickert RR, Auerbach O, Garfinkel L, Hammond EC, Frasca JM. Adenomatous lesions of the large bowel: an autopsy survey. Cancer. 1979;43(5):1847-57.

14. Vatn MH, Stalsberg H. The prevalence of polyps of the large intestine in Oslo: an autopsy study. Cancer. 1982;49(4):819-25.

15. Williams AR, Balasooriya BA, Day DW. Polyps and cancer of the large bowel: a necropsy study in Liverpool. Gut. 1982;23(10):835-42.

16. Stoop EM, de Haan MC, de Wijkerslooth TR, Bossuyt PM, van Ballegooijen M, Nio CY, et al. Participation and yield of colonoscopy versus non-cathartic CT colonography in population-based screening for colorectal cancer: a randomised controlled trial. Lancet Oncol. 2012;13(1):55-64.

17. Hardcastle JD, Chamberlain JO, Robinson MH, Moss SM, Amar SS, Balfour TW, et al. Randomised controlled trial of faecal-occult-blood screening for colorectal cancer. The Lancet. 1996;348(9040):1472-7.

18. Mandel JS, Church TR, Ederer F, Bond JH. Colorectal cancer mortality: effectiveness of biennial screening for fecal occult blood. J Natl Cancer Inst. 1999;91(5):434-7.

19. Jorgensen OD, Kronborg O, Fenger C. A randomised study of screening for colorectal cancer using faecal occult blood testing: results after 13 years and seven biennial screening rounds. Gut. 2002;50(1):29-32.

20. Kronborg O, Fenger C, Olsen J, Jorgensen OD, Sondergaard O. Randomised study of screening for colorectal cancer with faecal-occult-blood test. The Lancet. 1996;348(9040):1467-71.

21. Warren JL, Klabunde CN, Mariotto AB, Meekins A, Topor M, Brown ML, et al. Adverse events after outpatient colonoscopy in the Medicare population. Ann Intern Med. 2009;150(12):849-57, W152.

22. Gatto NM, Frucht H, Sundararajan V, Jacobson JS, Grann VR, Neugut AI. Risk of perforation after colonoscopy and sigmoidoscopy: a population-based study. J Natl Cancer Inst. 2003;95(3):230-6.

23. van Hees F, Zauber AG, Klabunde CN, Goede SL, Lansdorp-Vogelaar I, van Ballegooijen M. The appropriateness of more intensive colonoscopy screening than recommended in Medicare beneficiaries: a modeling study. JAMA Intern Med. 2014;174(10):1568-76.

24. Ness RM, Holmes AM, Klein R, Dittus R. Utility valuations for outcome states of colorectal cancer. Am J Gastroenterol. 1999;94(6):1650-7.

25. Gong YM, Peng P, Bao PP, Zhong WJ, Shi Y, Gu K, et al. The Implementation and First-Round Results of a Community-Based Colorectal Cancer Screening Program in Shanghai, China. Oncologist. 2018;23(8):928-35. DOI: 10.1634/theoncologist.2017-0451

26. Zhonghua Yi Xue Hui Xiao Hua Nei Jing Xue Fen Hui [Chinese Society of Digestive Endoscopy of the Chinese Medical Association], Zhongguo Kang Ai Xie Hui Zhong Liu Nei Jing Xue Zhuan Ye Wei Yuan Hui [The Society of Tumor Endoscopy of the Chinese Anti-Cancer Association]. Zhongguo Zao Qi Jie Zhi Chang Ai Shai Cha Ji Nei Jing Zhen Zhi Zhi Nan (Beijing, 2014)]. [Chinese guideline on the screening and endoscopic management of early colorectal cancer (Beijing, 2014)]. Wei Chang Bing Xue [Chin J Gastroenterol]. 2015;20(6):345-365. DOI: 10.3969/j.issn.1008-7125.2015.06.006
