# Supplementary material for: Cost-effectiveness analysis of colorectal cancer screening in Shanghai, China: A modelling study
Source: Prev Med Rep. 2022 Jul 4;29:101891. doi: 10.1016/j.pmedr.2022.101891 (PMC9294625; doi:10.1016/j.pmedr.2022.101891)
Supplement: Supplementary data 2 [file mmc2.docx]

Supplementary Results Figures

[Figures 2](#_Toc93685951)

[Figure S1: Costs and life years gained (undiscounted) per 1,000 simulated individuals for screening strategies. 2](#_Toc93685952)

[Figure S2: Costs and life years gained (discounted at 5%) per 1,000 simulated individuals for screening strategies. 3](#_Toc93685953)

[Figure S3: Costs and life years gained (discounted at 3%) per 1,000 simulated individuals for screening strategies. 4](#_Toc93685954)

[a. Assuming Chinese surveillance. 4](#_Toc93685955)

[b. Assuming treatment costs proportional to Australian treatment costs 5](#_Toc93685956)

[c. Assuming a 50% reduction in the costs of the validated FIT. 5](#_Toc93685957)

[d. Assuming a 200% increase in the costs of the validated FIT. 6](#_Toc93685958)

[e. Using data obtained from the other region in China, Guangzhou. 6](#_Toc93685959)

[f. Assuming increased participation for screening and diagnostic follow-up. 7](#_Toc93685960)

[g. Assuming international quality of life estimates 7](#_Toc93685961)

[Figure S4: Cost-effectiveness acceptability curve from the probabilistic sensitivity analysis. 8](#_Toc93685962)

## Figures

### Figure S1: Costs and life years gained (undiscounted) per 1,000 simulated individuals for screening strategies.

Abbreviations: FIT, faecal immunochemical test; LYs, life years; RA, risk assessment.

### Figure S2: Costs and life years gained (discounted at 5%) per 1,000 simulated individuals for screening strategies.

Abbreviations: FIT, faecal immunochemical test; LYs, life years; RA, risk assessment.

### Figure S3: Costs and life years gained (discounted at 3%) per 1,000 simulated individuals for screening strategies.

### Assuming Chinese surveillance.

Abbreviations: FIT, faecal immunochemical test; LYs, life years; RA, risk assessment.

### Assuming treatment costs proportional to lifetime health care costs for different stages from Lang et al. (1)

Abbreviations: FIT, faecal immunochemical test; QALYs, quality-adjusted life years; RA, risk assessment.

### Assuming a 50% reduction in the costs of the validated FIT.

Abbreviations: FIT, faecal immunochemical test; LYs, life years; RA, risk assessment.

### Assuming a 200% increase in the costs of the validated FIT.

Abbreviations: FIT, faecal immunochemical test; LYs, life years; RA, risk assessment.

### Using data obtained from the other region in China, Guangzhou.

Abbreviations: FIT, faecal immunochemical test; LYs, life years; RA, risk assessment.

### Assuming increased participation for screening and diagnostic follow-up.

Abbreviations: FIT, faecal immunochemical test; LYs, life years; RA, risk assessment.

### Assuming international quality of life estimates

Abbreviations: FIT, faecal immunochemical test; QALYs, quality-adjusted life years; RA, risk assessment.

### Figure S4: Cost-effectiveness acceptability curve from the probabilistic sensitivity analysis.


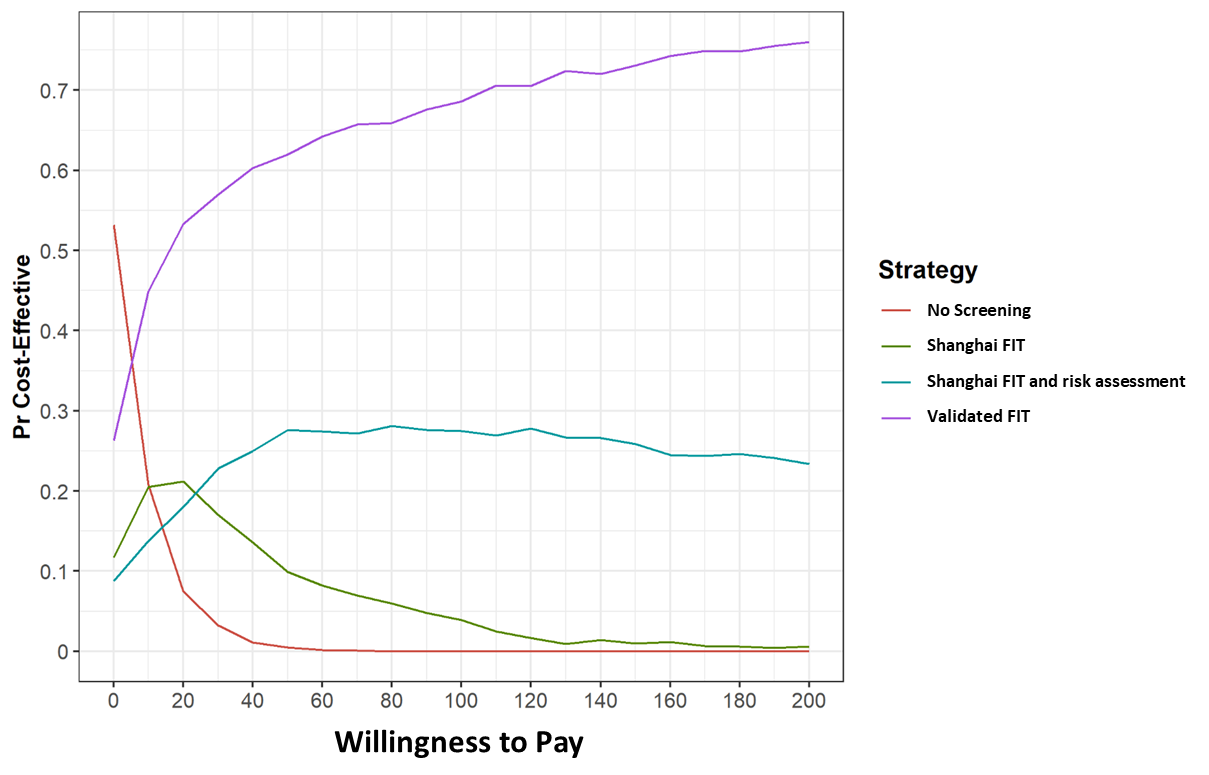


References:

1. Lang K, Lines LM, Lee DW, Korn JR, Earle CC, Menzin J. Lifetime and treatment-phase costs associated with colorectal cancer: evidence from SEER-Medicare data. Clinical Gastroenterology and Hepatology. 2009;7(2):198-204.
