# Supplementary material for: Cost-effectiveness analysis of colorectal cancer screening in Shanghai, China: A modelling study
Source: Prev Med Rep. 2022 Jul 4;29:101891. doi: 10.1016/j.pmedr.2022.101891 (PMC9294625; doi:10.1016/j.pmedr.2022.101891)
Supplement: Supplementary data 3 [file mmc3.docx]

Supplementary Results Tables

[Tables 2](#_Toc94013427)

[Table S1: Costs and effects (undiscounted) per 1,000 simulated individuals for screening strategies. 2](#_Toc94013428)

[Table S2: Costs and effects (discounted at 5%) per 1,000 simulated individuals for screening strategies. 2](#_Toc94013429)

[Table S3: Costs and effects (discounted at 3%) per 1,000 simulated individuals for screening strategies. 3](#_Toc94013430)

[a. Assuming a 50% reduction in the costs of the validated FIT. 3](#_Toc94013431)

[b. Assuming treatment costs proportional to lifetime health care costs for different stages from Lang et al. (REF) 3](#_Toc94013432)

[c. Assuming international quality of life estimates. 4](#_Toc94013433)

[d. Assuming Chinese surveillance. 4](#_Toc94013434)

[e. Using data obtained from the other region in China, Guangzhou. 5](#_Toc94013435)

[f. Assuming a 200% increase in the costs of the validated FIT. 5](#_Toc94013436)

[g. Assuming increased participation for screening (60%) and diagnostic follow-up (80%). 6](#_Toc94013437)

##

## Tables

### Table S1: Costs and effects (undiscounted) per 1,000 simulated individuals for screening strategies.

| **Screening strategy** | **Primary screening test episodes^a^** | **Colonoscopies** | **False Positives** | **Complications^b^** | **CRC Incidence** | **CRC Mortality** | **Life Years Gained^c^** | **Total Costs (¥)** | **ICER** |
| --- | --- | --- | --- | --- | --- | --- | --- | --- | --- |
| No Screening | 0 | 45 | 0 | 0.01 | 45 | 10 |  | 1,709,594 |  |
| Shanghai FIT | 2,145 | 171 | 56 | 0.01 | 43 | 9 | 12.71 | 1,763,916 | 4,274 |
| Shanghai FIT+RA | 2,142 | 197 | 70 | 0.01 | 42 | 9 | 13.63 | 1,788,403 | Dominated |
| Validated FIT | 2,150 | 122 | 14 | 0.01 | 43 | 9 | 14.19 | 1,792,411 | 19,253 |

Note: Grey shading highlights the most efficient screening strategy under the willingness-to-pay threshold.

Abbreviations: CRC, colorectal cancer; FIT, faecal immunochemical test; ICER, incremental cost-effectiveness ratio; RA, risk assessment.a. Shanghai FIT+RA and the validated two sample FIT were both considered to be one single test episode in the simulation.

b. Due to rounding and the low probability of an adverse event (complication) during a colonoscopy (0.012%), the number of complications per 1,000 individuals simulated did not change between different screening strategies.

c. Life years gained compared to a situation without screening.

### Table S2: Costs and effects (discounted at 5%) per 1,000 simulated individuals for screening strategies.

| **Screening strategy** | **Primary screening test episodes^a^** | **Colonoscopies** | **False Positives** | **Complications^b^** | **CRC Incidence** | **CRC Mortality** | **Life Years Gained^c^** | **Total Costs (¥)** | **ICER** |
| --- | --- | --- | --- | --- | --- | --- | --- | --- | --- |
| No Screening | 0 | 45 | 0 | 0.01 | 45 | 10 |  | 835,560 |  |
| Shanghai FIT | 2,145 | 171 | 56 | 0.01 | 43 | 9 | 4.01 | 885,380 | Dominated |
| Shanghai FIT+RA | 2,142 | 197 | 70 | 0.01 | 42 | 9 | 4.28 | 899,138 | Dominated |
| Validated FIT | 2,150 | 122 | 14 | 0.01 | 43 | 9 | 4.54 | 903,552 | 14,976 |

Note: Grey shading highlights the most efficient screening strategy under the willingness-to-pay threshold.

Abbreviations: CRC, colorectal cancer; FIT, faecal immunochemical test; ICER, incremental cost-effectiveness ratio; RA, risk assessment.

a. Shanghai FIT+RA and the validated two sample FIT were both considered to be one single test episode in the simulation.

b. Due to rounding and the low probability of an adverse event (complication) during a colonoscopy (0.012%), the number of complications per 1,000 individuals simulated did not change between different screening strategies.

c. Life years gained compared to a situation without screening.

### Table S3: Costs and effects (discounted at 3%) per 1,000 simulated individuals for screening strategies.

### Assuming a 50% reduction in the costs of the validated FIT.

| **Screening strategy** | **Primary screening test episodes^a^** | **Colonoscopies** | **False Positives** | **Complications^b^** | **CRC Incidence** | **CRC Mortality** | **Life Years Gained^c^** | **Total Costs (¥)** | **ICER** |
| --- | --- | --- | --- | --- | --- | --- | --- | --- | --- |
| No Screening | 0 | 45 | 0 | 0.01 | 45 | 10 |  | 1,080,042 |  |
| Validated FIT | 2,150 | 122 | 14 | 0.01 | 43 | 9 | 6.97 | 1,110,696 | 4,398 |
| Shanghai FIT | 2,145 | 171 | 56 | 0.01 | 43 | 9 | 6.19 | 1,129,839 | Dominated |
| Shanghai FIT+RA | 2,142 | 197 | 70 | 0.01 | 42 | 9 | 6.62 | 1,146,176 | Dominated |

Note: Grey shading highlights the most efficient screening strategy under the willingness-to-pay threshold.

Abbreviations: CRC, colorectal cancer; FIT, faecal immunochemical test; ICER, incremental cost-effectiveness ratio.

a. Shanghai FIT+RA and the validated two sample FIT were both considered to be one single test episode in the simulation.

b. Due to rounding and the low probability of an adverse event (complication) during a colonoscopy (0.012%), the number of complications per 1,000 individuals simulated did not change between different screening strategies.

c. Life years gained compared to a situation without screening.

### Assuming treatment costs proportional to lifetime health care costs for different stages from Lang et al. (1)

| **Screening strategy** | **Primary screening test epidsodes^a^** | **Colonoscopies** | **False Positives** | **Complications^b^** | **CRC Incidence** | **CRC Mortality** | **Life Years Gained^c^** | **Total Costs (¥)** | **ICER** |
| --- | --- | --- | --- | --- | --- | --- | --- | --- | --- |
| No Screening | 0 | 45 | 0 | 0.01 | 45 | 10 | 0.00 | 2,851,664 | Dominated |
| Validated FIT | 2,150 | 122 | 14 | 0.01 | 43 | 9 | 6.97 | 2,617,199 |  |
| Shanghai FIT and risk assessment | 2,142 | 197 | 70 | 0.01 | 42 | 9 | 6.62 | 2,647,939 | Dominated |
| Shanghai FIT | 2,145 | 171 | 56 | 0.01 | 43 | 9 | 6.19 | 2,662,620 | Dominated |

Note: Grey shading highlights the most efficient screening strategy under the willingness-to-pay threshold.

Abbreviations: CRC, colorectal cancer; FIT, faecal immunochemical test; ICER, incremental cost-effectiveness ratio.

a. Shanghai FIT+RA and the validated two sample FIT were both considered to be one single test episode in the simulation.

b. Due to rounding and the low probability of an adverse event (complication) during a colonoscopy (0.012%), the number of complications per 1,000 individuals simulated did not change between different screening strategies.

c. Life years gained compared to a situation without screening.

### Assuming international quality of life estimates.

| **Screening strategy** | **Primary screening test episodes^a^** | **Colonoscopies** | **False Positives** | **Complications^b^** | **CRC Incidence** | **CRC Mortality** | **Total QALYs** | **Total Costs (¥)** | **ICER** |
| --- | --- | --- | --- | --- | --- | --- | --- | --- | --- |
| No Screening | 0 | 45 | 0 | 0.01 | 45 | 10 | 13,170 | 1,080,042 |  |
| Shanghai FIT | 2,145 | 171 | 56 | 0.01 | 43 | 9 | 13,283 | 1,129,839 | 441 |
| Shanghai FIT+RA | 2,142 | 197 | 70 | 0.01 | 42 | 9 | 13,298 | 1,146,176 | Dominated |
| Validated FIT | 2,150 | 122 | 14 | 0.01 | 43 | 9 | 13,306 | 1,150,479 | 907 |

Note: Grey shading highlights the most efficient screening strategy under the willingness-to-pay threshold.

Abbreviations: CRC, colorectal cancer; FIT, faecal immunochemical test; ICER, incremental cost-effectiveness ratio; QALY, quality-adjusted life-years; RA, risk assessment.

a. Shanghai FIT+RA and the validated two sample FIT were both considered to be one single test episode in the simulation.

b. Due to rounding and the low probability of an adverse event (complication) during a colonoscopy (0.012%), the number of complications per 1,000 individuals simulated did not change between different screening strategies.

### Assuming Chinese surveillance.

| **Screening strategy** | **Primary screening test episodes^a^** | **Colonoscopies** | **False Positives** | **Complications^b^** | **CRC Incidence** | **CRC Mortality** | **Life Years Gained^c^** | **Total Costs (¥)** | **ICER** |
| --- | --- | --- | --- | --- | --- | --- | --- | --- | --- |
| No Screening | 0 | 45 | 0 | 0.01 | 45 | 10 |  | 1,080,042 |  |
| Shanghai FIT | 2,273 | 174 | 61 | 0.01 | 43 | 9 | 6.05 | 1,133,597 | 8,852 |
| Shanghai FIT+RA | 2,301 | 202 | 77 | 0.01 | 43 | 9 | 6.48 | 1,151,197 | Dominated |
| Validated FIT | 2,202 | 111 | 17 | 0.01 | 43 | 9 | 6.71 | 1,152,065 | 27,982 |

Note: Grey shading highlights the most efficient screening strategy under the willingness-to-pay threshold.

Abbreviations: CRC, colorectal cancer; FIT, faecal immunochemical test; ICER, incremental cost-effectiveness ratio.

a. Shanghai FIT+RA and the validated two sample FIT were both considered to be one single test episode in the simulation.

b. Due to rounding and the low probability of an adverse event (complication) during a colonoscopy (0.012%), the number of complications per 1,000 individuals simulated did not change between different screening strategies.

c. Life years gained compared to a situation without screening.

### Using data obtained from the other region in China, Guangzhou.

| **Screening strategy** | **Primary screening test episodes^a^** | **Colonoscopies** | **False Positives** | **Complications^b^** | **CRC Incidence** | **CRC Mortality** | **Life Years Gained^c^** | **Total Costs (¥)** | **ICER** |
| --- | --- | --- | --- | --- | --- | --- | --- | --- | --- |
| No Screening | 0 | 45 | 0 | 0.01 | 45 | 10 |  | 1,080,042 |  |
| Shanghai FIT | 2,152 | 99 | 21 | 0.01 | 44 | 10 | 4.20 | 1,129,245 | Dominated |
| Shanghai FIT+RA | 2,148 | 131 | 42 | 0.01 | 44 | 10 | 4.26 | 1,145,271 | Dominated |
| Validated FIT | 2,150 | 122 | 14 | 0.01 | 43 | 9 | 6.97 | 1,150,479 | 10,106 |

Note: Grey shading highlights the most efficient screening strategy under the willingness-to-pay threshold.

Abbreviations: CRC, colorectal cancer; FIT, faecal immunochemical test; ICER, incremental cost-effectiveness ratio.

a. Shanghai FIT+RA and the validated two sample FIT were both considered to be one single test epidsode in the simulation.

b. Due to rounding and the low probability of an adverse event (complication) during a colonoscopy (0.012%), the number of complications per 1,000 individuals simulated did not change between different screening strategies.

c. Life years gained compared to a situation without screening.

### Assuming a 200% increase in the costs of the validated FIT.

| **Screening strategy** | **Primary screening test episodes^a^** | **Colonoscopies** | **False Positives** | **Complications^b^** | **CRC Incidence** | **CRC Mortality** | **Life Years Gained^c^** | **Total Costs (¥)** | **ICER** |
| --- | --- | --- | --- | --- | --- | --- | --- | --- | --- |
| No Screening | 0 | 45 | 0 | 0.01 | 45 | 10 |  | 1,080,042 |  |
| Shanghai FIT | 2,145 | 171 | 56 | 0.01 | 43 | 9 | 6.19 | 1,129,839 | 8,045 |
| Shanghai FIT+RA | 2,142 | 197 | 70 | 0.01 | 42 | 9 | 6.62 | 1,146,176 | 37,993 |
| Validated FIT | 2,150 | 122 | 14 | 0.01 | 43 | 9 | 6.97 | 1,230,045 | 239,626 |

Note: Grey shading highlights the most efficient screening strategy under the willingness-to-pay threshold.

Abbreviations: CRC, colorectal cancer; FIT, faecal immunochemical test; ICER, incremental cost-effectiveness ratio.

a. Shanghai FIT+RA and the validated two sample FIT were both considered to be one single test episode in the simulation.

b. Due to rounding and the low probability of an adverse event (complication) during a colonoscopy (0.012%), the number of complications per 1,000 individuals simulated did not change between different screening strategies.

c. Life years gained compared to a situation without screening.

### Assuming increased participation for screening (60%) and diagnostic follow-up (80%).

| **Screening strategy** | **Primary screening tests^a^** | **Colonoscopies** | **False Positives** | **Complications^b^** | **CRC Incidence** | **CRC Mortality** | **Life Years Gained^c^** | **Total Costs (¥)** | **ICER** |
| --- | --- | --- | --- | --- | --- | --- | --- | --- | --- |
| No Screening | 0 | 45 | 0 | 0.01 | 45 | 10 |  | 1,080,042 |  |
| Validated FIT | 2,964 | 246 | 45 | 0.02 | 38 | 7 | 18.20 | 1,131,164 | 2,809 |
| Shanghai FIT | 2,849 | 436 | 186 | 0.03 | 38 | 7 | 17.30 | 1,150,298 | Dominated |
| Shanghai FIT+RA | 2,729 | 635 | 301 | 0.03 | 35 | 6 | 22.17 | 1,184,922 | 13,541 |

Note: Grey shading highlights the most efficient screening strategy under the willingness-to-pay threshold.

Abbreviations: CRC, colorectal cancer; FIT, faecal immunochemical test; ICER, incremental cost-effectiveness ratio.

a. Shanghai FIT+RA and the validated two sample FIT were both considered to be one single test episode in the simulation.

b. Due to rounding and the low probability of an adverse event (complication) during a colonoscopy (0.012%), the number of complications per 1,000 individuals simulated did not change between different screening strategies.

c. Life years gained compared to a situation without screening.

**Reference**:

1. 1. Lang K, Lines LM, Lee DW, Korn JR, Earle CC, Menzin J. Lifetime and treatment-phase costs associated with colorectal cancer: evidence from SEER-Medicare data. Clinical Gastroenterology and Hepatology. 2009;7(2):198-204.
